# Active human full-length CDKL5 produced in the Antarctic bacterium *Pseudoalteromonas haloplanktis* TAC125

**DOI:** 10.1186/s12934-022-01939-6

**Published:** 2022-10-14

**Authors:** Andrea Colarusso, Concetta Lauro, Marzia Calvanese, Ermenegilda Parrilli, Maria Luisa Tutino

**Affiliations:** 1 Department of Chemical Sciences, “Federico II” University of Naples, Complesso Universitario Monte S. Angelo-Via Cintia, 80126 Naples, Italy; 2 Istituto Nazionale Biostrutture e Biosistemi-I.N.B.B., Viale Medaglie d’Oro, 305-00136 Rome, Italy

**Keywords:** *Pseudoalteromonas haloplanktis* TAC125, Antarctic bacterium, Psychrophilic gene expression system, Intrinsically disordered protein (IDP), Bicistronic design, In cellulo kinase assay, Tricistronic design, Recombinant protein aggregation, Recombinant protein condensation

## Abstract

**Background:**

A significant fraction of the human proteome is still inaccessible to in vitro studies since the recombinant production of several proteins failed in conventional cell factories. Eukaryotic protein kinases are difficult-to-express in heterologous hosts due to folding issues both related to their catalytic and regulatory domains. Human CDKL5 belongs to this category. It is a serine/threonine protein kinase whose mutations are involved in CDKL5 Deficiency Disorder (CDD), a severe neurodevelopmental pathology still lacking a therapeutic intervention. The lack of successful CDKL5 manufacture hampered the exploitation of the otherwise highly promising enzyme replacement therapy. As almost two-thirds of the enzyme sequence is predicted to be intrinsically disordered, the recombinant product is either subjected to a massive proteolytic attack by host-encoded proteases or tends to form aggregates. Therefore, the use of an unconventional expression system can constitute a valid alternative to solve these issues.

**Results:**

Using a multiparametric approach we managed to optimize the transcription of the *CDKL5* gene and the synthesis of the recombinant protein in the Antarctic bacterium *Pseudoalteromonas haloplanktis* TAC125 applying a bicistronic expression strategy, whose generalization for recombinant expression in the cold has been here confirmed with the use of a fluorescent reporter. The recombinant protein largely accumulated as a full-length product in the soluble cell lysate. We also demonstrated for the first time that full-length CDKL5 produced in Antarctic bacteria is catalytically active by using two independent assays, making feasible its recovery in native conditions from bacterial lysates as an active product, a result unmet in other bacteria so far. Finally, the setup of an in cellulo kinase assay allowed us to measure the impact of several CDD missense mutations on the kinase activity, providing new information towards a better understanding of CDD pathophysiology.

**Conclusions:**

Collectively, our data indicate that *P. haloplanktis* TAC125 can be a valuable platform for both the preparation of soluble active human CDKL5 and the study of structural–functional relationships in wild type and mutant CDKL5 forms. Furthermore, this paper further confirms the more general potentialities of exploitation of Antarctic bacteria to produce “intractable” proteins, especially those containing large intrinsically disordered regions.

**Supplementary Information:**

The online version contains supplementary material available at 10.1186/s12934-022-01939-6.

## Background

Cyclin-dependent kinase-like 5 (CDKL5) is a serine/threonine protein kinase involved in the development of the human brain. Dozens of mutations of the *CDKL5* gene are causative of CDKL5 Deficiency Disorder (CDD; OMIM 300203; 300672) [1, 2], a severe condition that is manifested with intellectual disability, autistic behavior, motor and visual impairments, infantile-onset refractory epilepsy, and many other symptoms [3]. Although no cure for CDD exists today, some studies proved that the restoration of CDKL5 activity through either protein or genetic intervention can revert CDD symptoms in mice and human models [4–6]. To clearly understand the applicability of these measures, however, a deeper knowledge of CDKL5 biology, its regulation, and the genotype-phenotype relationship of each CDD mutation should be acquired. For instance, we do not know whether some CDD mutations are dominant negative. If so, either enzyme replacement therapy (ERT) or gene addition may not be equally beneficial to all CDD patients. Another important question that should be addressed involves the levels of kinase activity that should be reached not to have a detrimental effect on CDD patients. Recently, the hyperphosphorylation of CDKL5 T169 has been demonstrated to induce the kinase hyperactivation which is a stress signal that triggers cell death during acute kidney injury [7]. This outcome would suggest that the presence and the abundance of post-translational modifications (PTMs) in CDKL5 preparations could significantly alter the therapeutic potential of a CDKL5-based ERT.

**Fig. 1:**
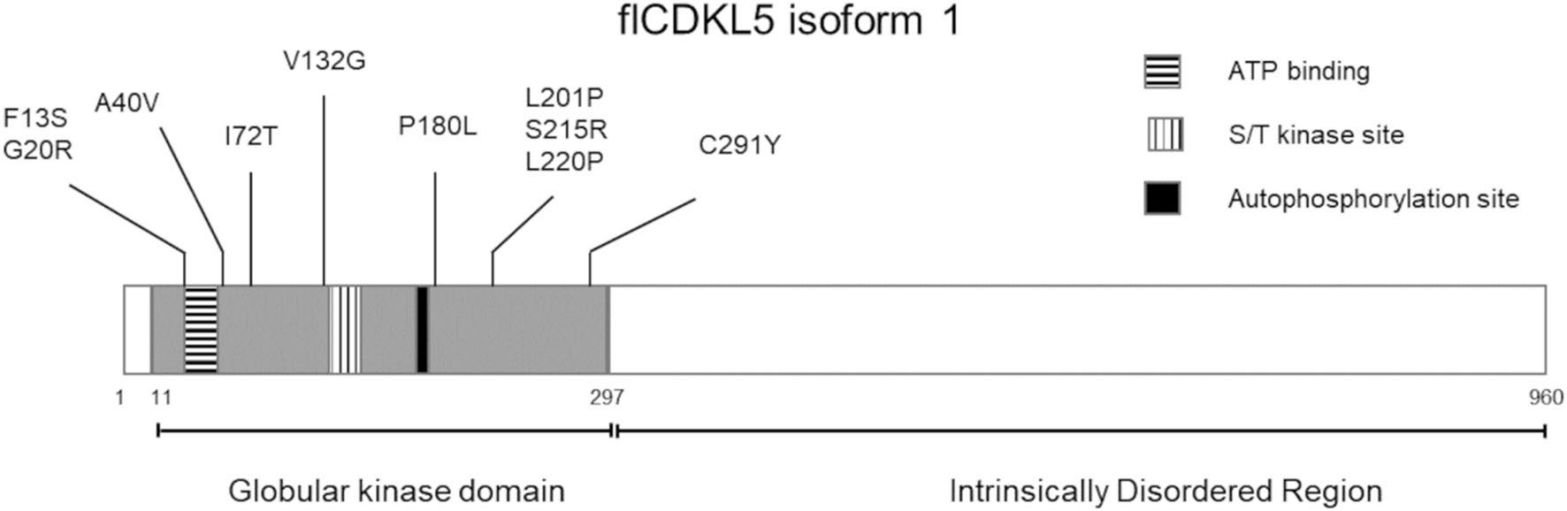
Scheme of the CDKL5 isoform 1 structural architecture and position of pathological mutations analyzed in this study. The grey box indicates the folded kinase domain harboring an ATP binding region a Serine/threonine (S/T) kinase active site and an autophosphorylation region. The remaining C-terminal extremity of the protein is predicted to be disordered

These observations indicate that multiple complementary tools to purify and study CDKL5 should be developed. Bacterial expression systems are desirable both for preparative and basic research purposes. First, the PTMs of the recombinant protein expressed in a bacterium would be limited to the phosphorylations dependent on the kinase autocatalytic activity, and a comparison with CDKL5 isolated from eukaryotic sources would make it possible to understand the role of other PTMs and their impact in an ERT. For similar reasons, a prokaryotic platform can be a valid tool to support the research in finding CDKL5 interactors, substrates, and upstream regulators. Different groups have already identified some CDKL5 substrates both in the cytoplasm [8, 9] and in the nucleus [10] using human cell cultures. Furthermore, a yeast two-hybrid screening has provided a list of proteins potentially interacting with the C-terminal extremity of CDKL5, although just two have been disclosed [11, 12]. We think that a reliable bacterial system can be useful to speed up the validation process of potential interactors and/or other putative substrates present in such lists. This aspect is crucial in the case of protein kinases since they generally can activate phosphorylation cascades and distinguishing each phosphorylation step from another can be troublesome in a eukaryotic context. In this regard, bacteria have often been useful in the study of eukaryotic kinases, from Cobb’s pioneering work about the reconstruction of a MAP kinase cascade in *Escherichia coli* [13] to more recent examples involving the characterization of the activation process of ciliary kinases through the comparison of protein preparations from different recombinant sources [14, 15]. The recombinant production of full-length CDKL5 (flCDKL5) is a challenging task, though. Its gene encodes a transcript that is subjected to alternative splicing leading to the production of five isoforms whose functional differences, if present, have not been explored [16]. However, the most abundant CDKL5 variant in the brain is isoform 1, followed by isoforms 2 and 3, and to a very little extent by isoform 4. Whereas isoform 5—which was the most studied one in early literature—is the bigger variant, but is exclusively expressed in the testis [16]. From a compositional point of view, all five isoforms are similar to each other since their cognate transcripts just differ for a few exons. For this reason, from now on we will generally refer to isoform 1 with the name flCDKL5. As recently highlighted by an in silico analysis and schematized in Fig. 1, flCDKL5 is predicted to be mainly a disordered protein, except for its N-terminal catalytic domain [17]. In support of this hypothesis, flCDKL5 has been recently demonstrated to interact with poly(ADP-ribose) with its C-terminal extremity [10], a typical feature of proteins possessing intrinsically disordered regions (IDRs) [18]. Proteins harboring extended IDRs are often problematic to be recombinantly expressed in and purified from conventional biotechnological platforms due to their propensity to either be degraded or to condensate [19]. flCDKL5 seems to fall in this category of difficult-to-express proteins. Even if its kinase catalytic domain has been successfully expressed in insect cells, yeast [20] and even *E. coli* [21], reports of recombinant production of flCDKL5 are quite scarce. Worth mentioning is the co-expression assay of flCDKL5 with two different substrates that Muñoz and co-workers established in HEK293 cells to evaluate the impact of CDD mutations [8]. Nevertheless, no examples of flCDKL5 satisfying expression in bacterial cells are available. The protein is insoluble in *E. coli* and denaturation and refolding procedures failed to achieve the active protein [22]. Regardless of this significant drawback, Katayama and Inazu managed to develop an ingenious assay to observe the flCDKL5 autophosphorylation activity on *E. coli* insoluble fractions, although such an approach would make it difficult to study the interaction of the kinase with other proteins [23]. As a result, a bacterial system that allows the soluble accumulation of flCDKL5 is desirable to allow both the validation of putative interactors with a co-expression assay and to obtain the active protein in native conditions after the cellular lysis.

To pursue this objective, we here present the genetic tools we have developed to produce flCDKL5 in the model Antarctic bacterium *Pseudoalteromonas haloplanktis* TAC125 (also named *P. translucida* TAC125). This prokaryote has already proven to be a useful alternative to conventional bacteria for the synthesis of difficult-to-express eukaryotic proteins [24–26]. The production of flCDKL5 isoform 5 has been tested in this bacterium but the overall yield was extremely low and the cultivation temperature of 0 °C was needed to preserve the protein during its synthesis [27]. However, very recently a model developed to study the *P. haloplanktis* TAC125 metabolism [28] has been specifically applied to optimize the production of flCDKL5 isoform 1, which is the most abundant in the human brain [29]. Here, we report the design of two sets of plasmids: the first set has been iteratively optimized to produce flCDKL5 isoform 1 fused with multiple tags for its detection and isolation; the second set has been designed to allow the co-expression of flCDKL5 with any one of its either putative or confirmed protein substrates. The former plasmid will be used to produce flCDKL5 for future applicative purposes, while the second will be used for basic research, e.g. either to study the effect of CDD mutations on flCDKL5 activity on known substrates or validate flCDKL5 putative interactors discovered with independent assays. As proof of concept, we here demonstrate that, by using the first set of plasmids, flCDKL5 can be produced mainly in intact and soluble form in *P. haloplanktis* TAC125 and that, after enrichment in native conditions, the kinase is catalytically active. Furthermore, by using the second set of plasmids, we co-expressed the 10 flCDKL5 CDD mutants shown in Fig. 1 with the CDKL5 bonafide protein substrate EB2 [8, 9] and demonstrated that this bacterial tool can effectively measure the impact of each mutation on the enzymatic activity. Our future goal is to use these two platforms to extend the knowledge of CDKL5 biology and to achieve protein purification for ERT. For this reason, all the constructs presented in this work possess an N-terminal TATκ peptide that has already been used to vehiculate flCDKL5 across the blood-brain barrier [4].

## Results and discussion

### A multiparametric strategy to optimize full-length CDKL5 expression in *Pseudoalteromonas haloplanktis* TAC125

**Fig. 2:**
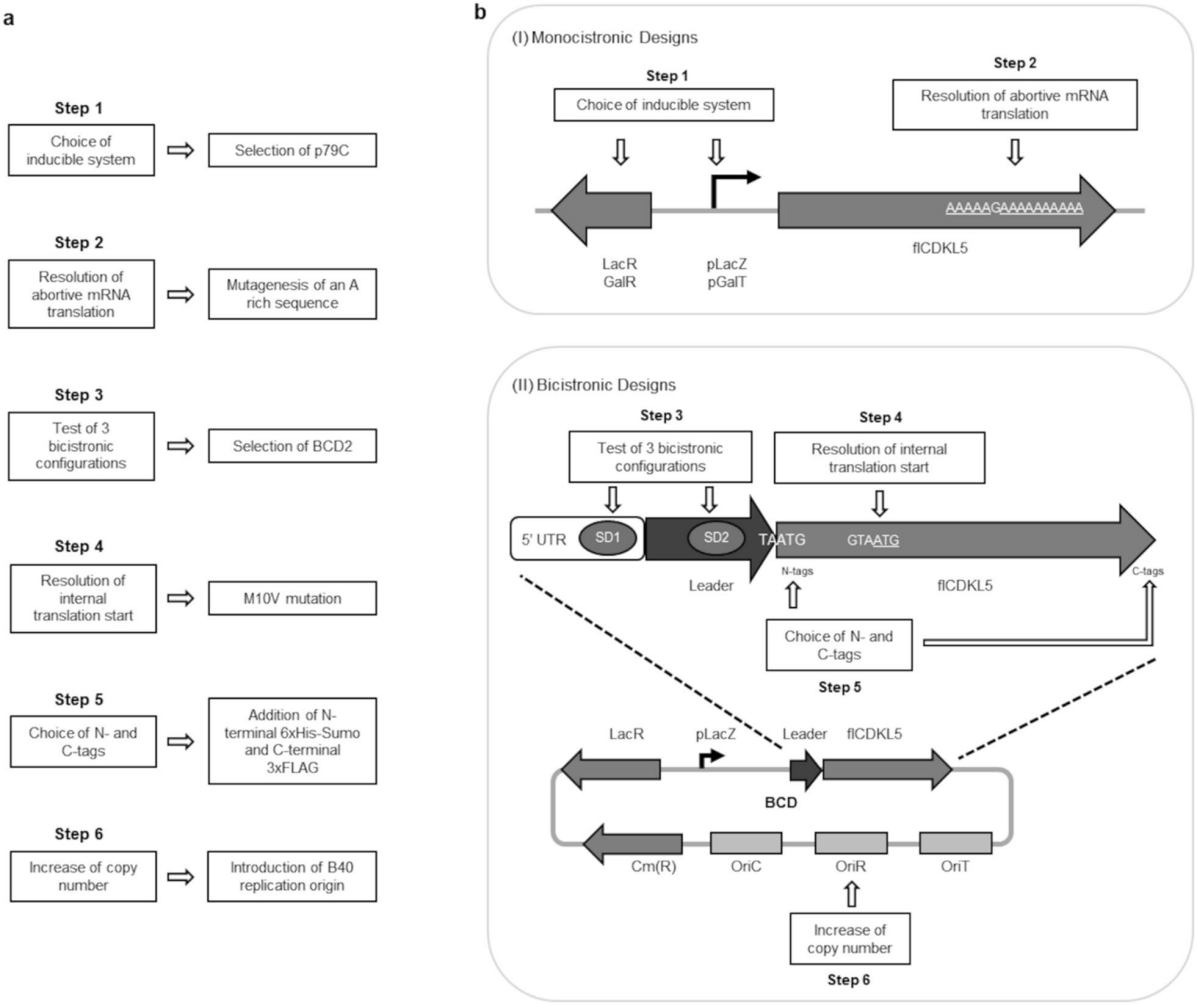
Development of bicistronic plasmids for the production of flCDKL5.** a** Stepwise optimization of the production of flCDKL5. **b** Schemes of Monocistronic (I) and Bicistronic (II) plasmids for the expression of flCDKL5. (I) In the monocistronic configurations we tested two inducible systems (Step 1): p79C dependent on the LacR/pLacZ couple; pMAV dependent on the GalR/pGalT couple. Furthermore, a mutagenesis approach was used to overcome a translation abortion due to a polyA sequence (AAAAAGAAAAAAAAAA, Step 2). (II) The Bicistronic Designs (BCDs) were developed by testing three different combinations of SD1, SD2 and Leader peptide encoding sequences (Step 3). Additionally, a methionine encoding sequence (GTAATG) was mutated to abolish an internal translation start (Step 4) and different permutations of N- and C-terminal tags were applied to increase the preservation and detectability of flCDKL5 extremities (Step 5). Finally, the protein yield was increased by replacing the parental plasmid replication origin with a new one named B40 (Step 6). LacR and pLacZ, the regulator and the promoter of the lacZ gene in *P. haloplanktis* TAE79, respectively; GalR and pGalT, the regulator and the promoter of the GalT gene in *P. haloplanktis* TAC125; 5’ UTR, 5’ untranslated region; SD1 and SD2, Shine Dalgarno 1 and 2, respectively; Leader, upstream sequence encoding a short peptide coupled to flCDKL5 translation through the overlap of a stop and a start codons (TAATG); Cm(R), chloramphenicol resistance marker; OriC, *E. coli* pMB1 replication origin; OriR, Antarctic replication origin; OriT, origin for plasmid conjugative transfer [33]. The underlined sequences were targeted for nucleotide substitution

To test if *P. haloplanktis* TAC125 can be used to both express full-length CDKL5 (flCDKL5) for preparative purposes and as an orthogonal system for studying flCDKL5 functions and disease-related alterations, the protein kinase was produced both alone and together with one of its bonafide substrates, EB2 [8, 9] so as to carry out an *in cellulo* kinase assay. To do so, we first optimized the production of flCDKL5 alone, and then we applied the acquired knowledge to set up the co-expression assay. As schematized in Fig. 2a, six major steps were faced to progressively improve the production of intact flCDKL5 alone.

First, to express flCDKL5 we tested *P. haloplanktis* TAC125 monocistronic low copy number plasmids that were already available [30, 31]. In this phase, we focused on the selection of the best inducible promoter (Step 1 and Fig. 2b, panel (I)) and the resolution of an unforeseen abortion of mRNA translation (Step 2).

In the second part of this work, flCDKL5 synthesis was tested in bicistronic plasmids (BCDs, BiCistronic Designs), exploiting the translational coupling of the gene of interest with an optimized upstream ORF to increase the rate of translation initiation (Fig. 2b, panel (II)) [32]. This approach was pursued to increase the mRNA translation efficiency and stability. In this phase, we screened different combinations of upstream ORFs (Leader in Fig. 2b) and Shine Dalgarno sequences upstream of the Leader sequence (SD1) and the flCDKL5 encoding sequence (SD2, Step 3). We also overcame an internal translation start (Step 4) and optimized the disposition of N- and C-terminal tags to preserve flCDKL5 integrity (Step 5). Finally, the overall yield of flCDKL5 was significantly increased by the replacement of the replication origin of the plasmid with a new one that guaranteed an increased copy number (Step 6).

#### flCDKL5 expression with monocistronic plasmids

To define whether flCDKL5 can be produced and is detectable in recombinant Antarctic bacteria, we started this work with a conventional monocistronic asset. The flCDKL5 gene was designed to encode the human CDKL5 isoform 1—also called 107 variant [16]—with multiple tags. It possessed an N-terminal TATκ peptide that can be exploited for intracellular delivery [4], and tandem 3xFLAG and 6xHis C-terminal tags. Codon composition was automatically optimized for *P. haloplanktis* TAC125 by using the OPTIMIZER tool with the guided random method [34]. This CDKL5 variant was named 107 (B) and its expression was attempted in *P. haloplanktis* TAC125 cells using either a D-galactose inducible (pMAV) [30] or an IPTG inducible plasmid (p79C) [31], harboring a weak and strong regulatable promoters, respectively [31]. Regardless of their different expression mechanisms, the two plasmids share a common backbone and, most notably, harbor the same replication origin (OriR) derived from the *P. haloplanktis* TAC125 endogenous plasmid pMtBL [33]. Since pMtBL was demonstrated to be stably inherited as a single copy plasmid in Antarctic cells thanks to its partition sequences [35], we checked for the plasmid copy number (PCN) and stability of the shuttle recombinant vector p79C which is devoid of such regions. As shown in Additional file 1: Fig. S1a, the recombinant plasmid was stably kept with an average of 2–3 PCN during a 40-hour culture at 15 °C when the antibiotic selection was applied. Furthermore, IPTG induction did not seem to cause any instability (Additional file 1: Fig. S1b). Once the plasmid stability had been ascertained, 107 (B) production in the Antarctic bacterium was analyzed. After recombinant induction, 107 (B) production was not noticeable in *P. haloplanktis* TAC125 using SDS-PAGE on total lysates derived from either pMAV- or p79C-mediated expression (3a, left panel).

**Fig. 3:**
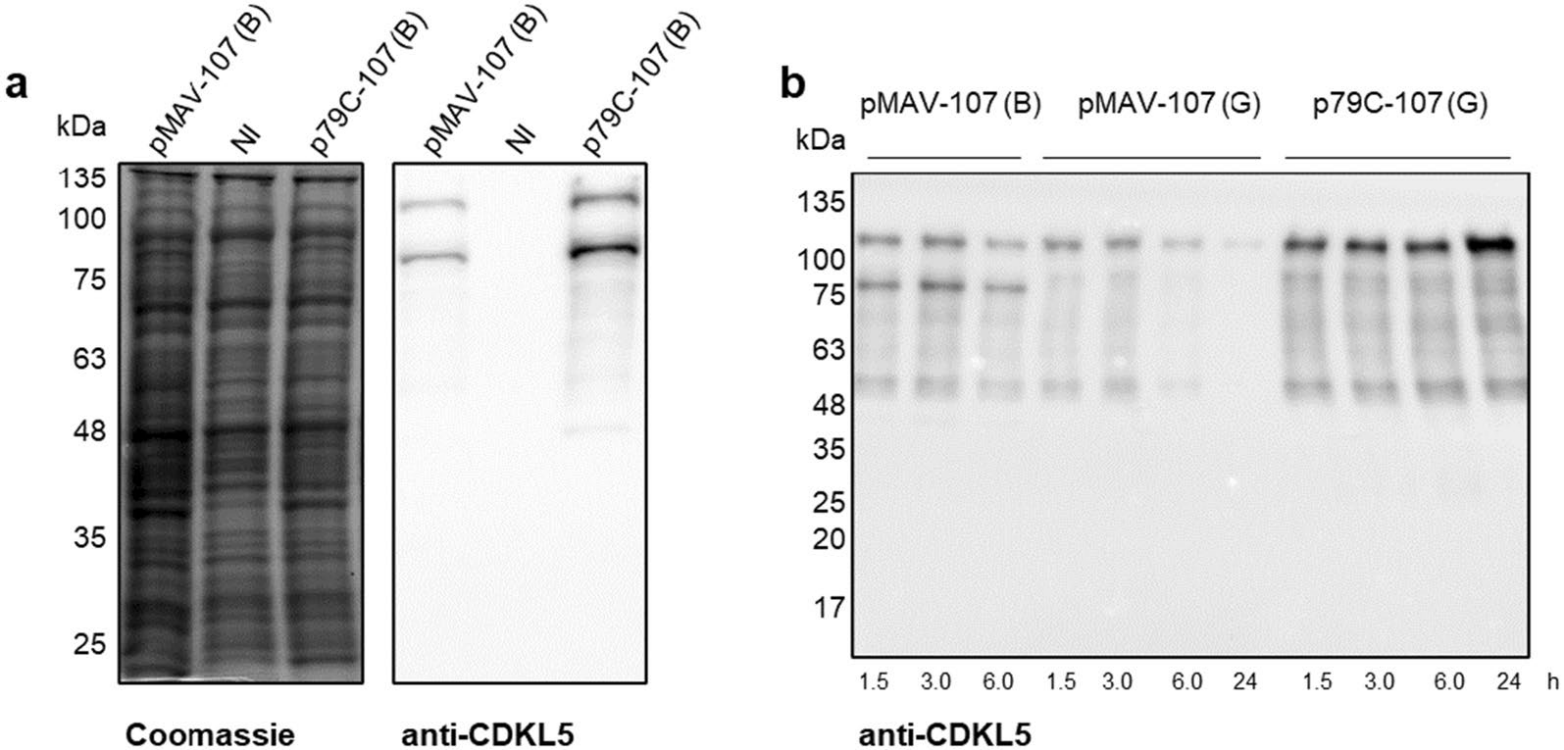
flCDKL5 production profile with pMAV and p79C monocistronic plasmids. **a** SDS-PAGE (left panel) and anti-CDKL5 Western blot (right panel) of *P. haloplanktis* TAC125 crude lysates after the expression of the flCDKL5 variant named 107 (B). Two plasmids were tested in comparison to a noninduced strain (NI): the D-galactose inducible plasmid pMAV and the IPTG-inducible plasmid p79C. **b** Comparison of the expression profile of 107 (B) with 107 (G). These flCDKL5 variants are identical in terms of residues composition, but their nucleotide sequences differ for the removal of an A-rich stretch which is causative of an abortive truncation when 107 (B) is translated. The time points indicated below refer to the hours after induction.

However, positive signals could be detected by anti-CDKL5 Western blot in both strains, proving that the target protein was synthesized, although at low levels (Fig. 3a, right panel). Furthermore, even if a signal compatible with flCDKL5 was visible in the Western blot analysis, a similarly intense band with a lower molecular weight was produced both by pMAV and p79C plasmids. Given that the used antibody targets the first half of the protein, this truncated product probably lacks the C-terminal extremity. To identify the molecular basis of this truncation, we analyzed the nucleotide sequence of the automatically codon-optimized 107 (B) gene and we noticed the occurrence of an A-rich region at about three-quarters (Fig. 2b, panel (I)). This polyA stretch corresponds to codons for six consecutive lysines, given that AAA is the most common triplet for this amino acid in *P. haloplanktis* TAC125. Since multiple authors have reported that A repetitions in mRNAs induce ribosome stalling, sliding, and accidental frameshift during protein synthesis [36–38], we modified this sequence by replacing five AAA codons with synonymous AAG. This new flCDKL5 variant was named 107 (G) and its expression profile was compared with the one of 107 (B). As shown in Fig. 3b, the main truncation product visible in the pMAV-107 (B) bearing strain was not produced by cells hosting either pMAV-107 (G) or p79C-107 (G). Furthermore, the IPTG inducible plasmid guaranteed a higher accumulation of the target protein over time than the D-galactose-dependent vector, a result that is in agreement with the different strengths of the two promoters [31]. For these reasons, we focused on the improvement of the p79C plasmid and the 107 (G) construct for further studies.

#### Bicistronic cassettes and site-specific mutagenesis to overcome an internal translational start

In the second phase of this study, we wanted to address two questions: can flCDKL5 production be increased by translational regulation? Is there an ideal disposition and combination of tags to preserve flCDKL5 extremities? As flCDKL5 is a high molecular weight protein (107 kDa) with extensive disordered regions [17], we cannot exclude possible either N- or C-terminal truncations just by electrophoretic migration. This is a non-trivial issue in the case of the here presented flCDKL5 variants since they are characterized by two flexible ends, the N-terminal TATκ and the C-terminal intrinsically disordered region (IDRs). IDRs can be a target of proteolysis and impact the half-life of proteins, but their fusion with tags can change their overall stability [39].

Hence, the second phase of our study aimed to examine and possibly improve these aspects contextually by using a technique that could both modulate translational efficiency and test different combinations of tags.

The tool we chose for this purpose is the bicistronic cassette (BCD) that introduces short optimized coding sequences upstream of the heterologous gene of interest to optimize the start of translation [32]. This approach has been used several times in different recombinant bacteria in the past and has demonstrated that the translation of a heterologous ORF can be modulated by the translational coupling with an upstream optimized short ORF. The application of this technology to finetune a psychrophilic translation system to produce a eukaryotic protein normally synthesized at 37 °C is reasonable given that the mRNA translation is differently regulated in bacteria and mammals [40] and that the temperature is likely to play a pivotal role in the kinetics of protein synthesis and folding. Given that protein synthesis is mainly limited by translation initiation and early elongation [41], we reasoned that the BCD strategy could serve as an insulator to optimize flCDKL5 synthesis so as to avoid too many interventions on the gene composition. Consequently, we implemented this technology in *P. haloplanktis* TAC125 using the work by Mutalik and coworkers as a model both for the design of the plasmids—as indicated in the Methods section—and for the nomenclature of the bicistronic designs [32]. Furthermore, the adopted cloning strategy allowed for the easy permutation of tags at the two extremities of flCDKL5 (see the Methods section). For these reasons BCD vectors were the most suitable tool to simultaneously analyze the effect of tags and translational control on flCDKL5 production and preservation.

The main components of a BCD cassette are: a 5’ untranslated region (5’ UTR) harboring a first SD sequence (SD1); a short open reading frame (ORF) that encodes a peptide optimized for initial translation (Leader sequence) and embeds a second SD sequence (SD2); the gene of interest whose translation driven by SD2 is coupled with the translation of the Leader sequence thanks to the overlap of a Stop and Start codons (Fig. 2b, panel (II)). The impact of variable BCD configurations on protein expression was assessed both using plasmids for the syntheses of a fluorescent reporter, pGFP [31], and a parallel set for the production of flCDKL5 (Additional file 1: Table S1 and Table 1, respectively).

**Table 1:** Characteristics of BCD constructs for flCDKL5 expression

Name	Leader^a^	SD1^b^	SD2^c^	N-tags^d^	C-tags^d^	Mutations^d^
p79C-107 (G)	/	/	CAACAGGAA	TATκ	3xFLAG-6xHis	/
pBCD1-107 (G)	LacZ	CAACAGGAA	CAACAGGAA	TATκ	3xFLAG-6xHis	/
pBCD2-107 (G)	LacZ	CAACAGGAA	AAGGAGGTC	TATκ	3xFLAG-6xHis	/
pBCD1-107 (K)	LacZ	CAACAGGAA	CAACAGGAA	6xHis-TATκ	3xFLAG	/
pBCD2-107 (K)	LacZ	CAACAGGAA	AAGGAGGTC	6xHis-TATκ	3xFLAG	/
pBCD3-107 (K)	TrpA	AAGGAGGTC	AAGGAGGTC	6xHis-TATκ	3xFLAG	/
pBCD2-107 (G) M10V	LacZ	CAACAGGAA	AAGGAGGTC	TATκ	3xFLAG-6xHis	M43V
pBCD2-107 (G) STOP	LacZ	CAACAGGAA	AAGGAGGTC	TATκ	3xFLAG-6xHis	M1I, G2STOP
pBCD2-107 (K) M10V	LacZ	CAACAGGAA	AAGGAGGTC	6xHis-TATκ	3xFLAG	M61V
pBCD2-107 (L) M10V	LacZ	CAACAGGAA	AAGGAGGTC	6xHis-Sumo-TATκ	3xFLAG	M159V

^a^Leader peptide encoded upstream of flCDKL5 includes the firs 19 residues of the indicated proteins. ^b^Shine Dalgarno sequence upstream of the Leader peptide encoding sequence. ^c^Shine Dalgarno sequence upstream of the flCDKL5 gene. Shine Dalgarno sequences which are entirely complementary to 16S rRNA are underlined. ^d^N- and C-terminal tags and mutations are referred to flCDKL5 protein. M43V, M61V and M159V are the same mutation (M10V in human CDKL5, O76039, Uniprot), but the coordinates differ because of the presence of different N-terminal tags

In the case of pGFP, we initially tested the first 19 residues of *P. haloplanktis* TAE79 LacZ as a Leader peptide, given the high expression levels of this psychrophilic β-galactosidase in *P. haloplanktis* TAC125 [31]. The two BCD vectors that were generated differed only for the SD2 sequence. In pBCD1, SD2 was the same as SD1, that is the natural SD sequence upstream of the lacZ ORF. In pBCD2, SD2 was optimized to embed a region that is perfectly complementary to the 3’-OH end of *P. haloplanktis* TAC125 16S rRNA (Additional file 1: Table S1). When the expression levels driven by these two bicistronic vectors were compared with a monocistronic cassette (p79C-pGFP), we could detect pGFP accumulation in all the cases, but pBCD2-pGFP guaranteed a significantly higher fluorescence suggesting that the optimized SD2 sequence was pivotal to increase the pGFP translation rate (Additional file 1: Fig. S2a). We also tested a different SD1—Leader combination to produce pGFP. We aimed to replace the theoretically weak SD of lacZ in position SD1 with an SD that is perfectly complementary to 16S rRNA by using an ORF that naturally possesses an optimized SD in *P. haloplanktis* TAC125 genome. The ORF we selected is in the *trpA* gene (PSHA_RS06350) of the tryptophan operon. The new plasmid was named pBCD3-H6-pGFP (Additional file 1: Table S1) and the levels of pGFP that could be accumulated were compared to pBCD1 and pBCD2 configurations. As visible in Additional file 1: Fig. S2a, although pBCD3 performed better than pBCD1, it was worse than pBCD2. This indicates that both SD1, SD2, and the Leader sequence play a role in the accumulation of the protein encoded by the second cistron. However, while the effect of SD2 seems predictable, more subtle variables may influence the roles of SD1 and the Leader cistron, as expected. The role of BCD constructs is to optimize the expression of the second cistron independently of its codon composition thanks to the insulating effect of the first cistron. On the other hand, the translation starting from the first cistron must be optimized with thorough screening, due to the lack of any insulator [32]. The addition of N-terminal 6xHis-TATκ tags did not significantly affect pGFP expression in the BCD2 configuration, indicating that multiple N-terminal tags can be added with no major detrimental effects on protein accumulation (Additional file 1: Fig. S2b). Overall, the data acquired with the fluorescent reporter indicate that BCD2 is the best bicistronic configuration we developed for recombinant expression in *P. haloplanktis* TAC125 and that the addition of N-terminal tags does not seem to perturb the pGFP production levels when this asset is used.

**Fig. 4:**
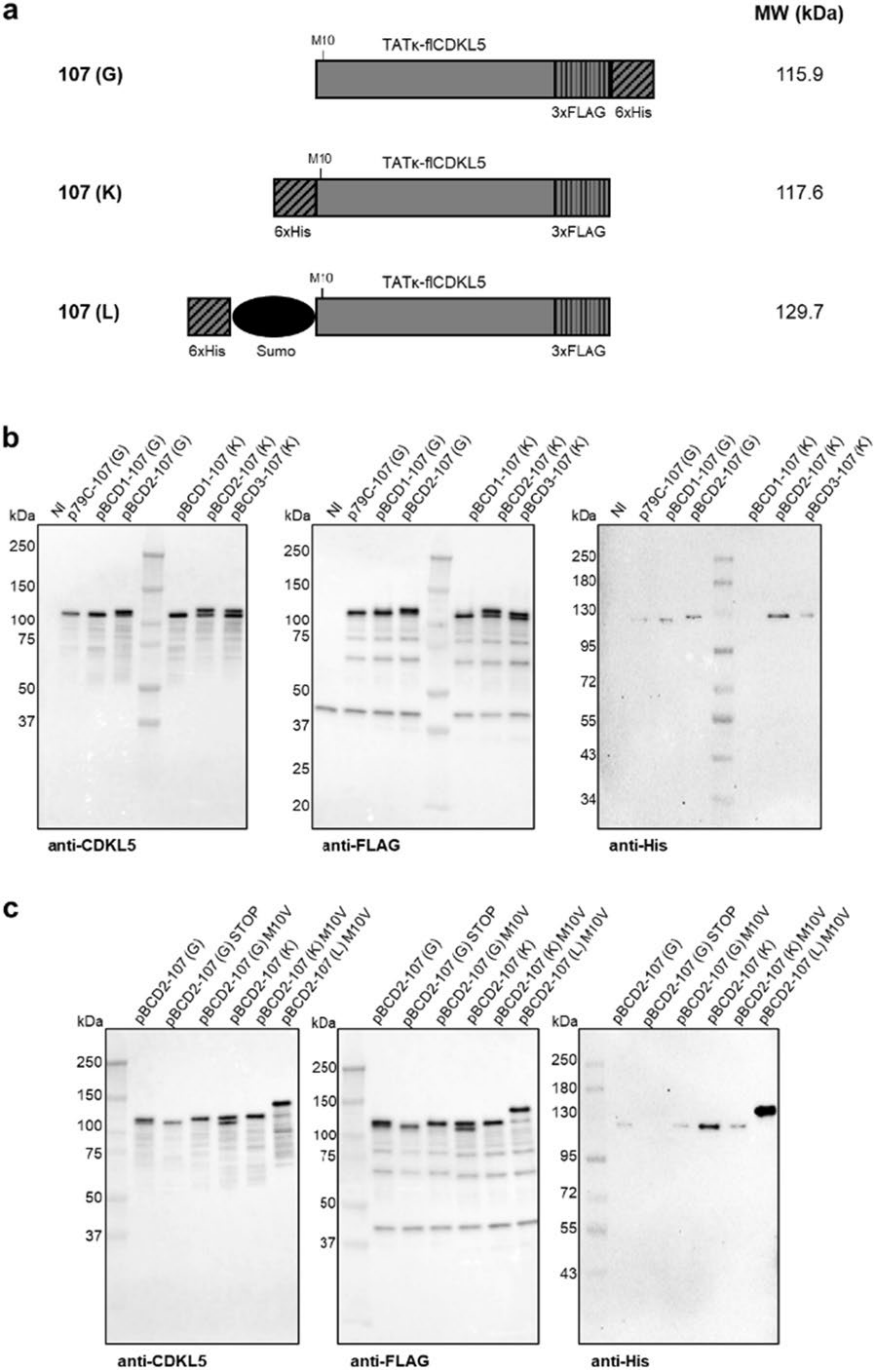
flCDKL5 production profiles with BCD plasmids.** a** Schemes of flCDKL5 constructs tested with BCD plasmids. All flCDKL5 variants are N-terminally fused to a TATκ peptide for intracellular vehiculation [4]. 107 (G) has C-terminal 3xFLAG and 6xHis tags. 107 (K) has an N-terminal 6xHis and a C-terminal 3xFLAG tag. 107 (L) has tandem N-terminal 6xHis and Sumo tags, and a 3xFLAG at the C-terminus. The residue M10 that was subjected to mutation is indicated for all flCDKL5 variants. **b** anti-CDKL5 (left panel), anti-FLAG (middle panel), and anti-His Western blots (right panel) of *P. haloplanktis* TAC125 crude lysates after the expression of the 107 (G) and 107 (K) variants in monocistronic (p79C) and BCD1, BCD2 and BCD3 bicistronic configurations. **c** anti-CDKL5 (left panel), anti-FLAG (middle panel), and anti-His Western blots (right panel) of *P. haloplanktis* TAC125 crude lysates expressing multiple either wt or mutant 107 (G), 107 (K), and 107 (L) variants with BCD2 plasmids. *NI* not induced strain

In the case of flCDKL5, the achievement of protein accumulation was less straightforward than pGFP. We tested the three bicistronic configurations (BCD1, BCD2, BCD3) that were explored for pGFP expression (Table 1). Furthermore, flCDKL5 was originally produced with two different dispositions of tags. The most relevant traits are that variant 107 (G) has C-terminal 3xFLAG and 6xHis, while 107 (K) harbors an N-terminal 6xHis and a C-terminal 3xFLAG tags (Table 1 and Fig. 4a). Anti-CDKL5 and anti-FLAG Western blots showed that pBCD2 and pBCD3 configurations gave rise to the production of a high molecular weight band that is not generated by either the monocistronic vector or pBCD1 (Fig. 4b, left and middle panels).

Apparently, p79C and pBCD1 plasmids only generated a truncated version of flCDKL5, while pBCD2 and pBCD3 allowed the synthesis of both the truncated and the putatively intact forms of flCDKL5 both as 107 (G) and 107 (K) variants. Given that such two bands are detected by the anti-FLAG antibody and that both 107 (G) and 107 (K) have a C-terminal 3xFLAG tag (Fig. 4a), the lower molecular weight band is probably N-terminally truncated. This hypothesis is also suggested by the anti-His Western blot that detected positive signals for all the strains but pBCD1-107 (K, Fig. 4b, right panel). 107 (G) was always reactive because the 6xHis tag is C-terminal in this flCDKL5 variant. On the other hand, the 107 (K) variant has an N-terminal 6xHis tag and the lack of any anti-His detection in the pBCD1-107 (K) bearing strain is indicative of the fact that in this condition flCDKL5 is exclusively synthesized in an N-terminally truncated form (detectable by anti-FLAG and anti-CDKL5 antibodies). Since the three BCD configurations just differ for elements regulating the start of flCDKL5 translation, we reasoned that such variable fragmentation patterns might be due to a translational bias. We formulated two hypotheses: (1) flCDKL5 was always N-terminally proteolyzed in *P. haloplanktis* TAC125, but BCD2 and BCD3 allowed for the accumulation of the intact form of the protein thanks to higher production levels (see Additional file 1: Fig. S2 for a comparison with pGFP accumulation); (2) the flCDKL5 encoding gene harbored an internal translation start that overrode the first translation start unless the latter is optimized like in the BCD2 and BCD3 configurations. A new analysis of the flCDKL5 encoding gene pointed toward the second hypothesis since it highlighted the possible existence of a cryptic internal translation initiation at the level of M10 according to the coordinates of the human CDKL5 isoform 1 (O76039, Uniprot). Although a strong Shine Dalgarno sequence could not be found upstream of the ATG encoding M10, a truncation at this level is compatible with the small electrophoretic difference between intact flCDKL5 and truncated flCDKL5 forms in our Western blot analysis (Fig. 4b).

To test this hypothesis of internal translation initiation, we planned multiple experiments. First, we designed a new flCDKL5 variant possessing a bulky tag between the N-terminal 6xHis and the CDKL5 wt residues to make the truncated product more easily distinguishable from intact CDKL5. This construct was named 107 (L) and harbors N-terminal 6xHis-Sumo tags and a C-terminal 3xFLAG (Fig. 4a). This modification served also to test our first hypothesis (i.e. whether flCDKL5 was truncated due to degradation) since the fusion of flexible extremities with globular domains can change their propensity to degradation, as already mentioned [39]. Then, we introduced site-directed mutations in 107 (G), 107 (K), and 107 (L) flCDKL5 variants to reveal the putative role of M10 as an internal translation start. In particular, we abolished the translation initiation from the first residue of 107 (G) by eliminating the first ATG and introducing a STOP codon in the second position (pBCD2-107 (G) STOP in Table 1). As revealed by anti-CDKL5 and anti-FLAG Western blots (Fig. 4c, left and middle panels, respectively), the truncated form of flCDKL5 was still produced by pBCD2-107 (G) STOP bearing cells, while the upper band was absent, as expected. This outcome suggests that an internal translation start occurred. Then, we replaced M10 of human CDKL5 with a valine to have an insight into its role in flCDKL5 fragmentation. Although such residue has different coordinates depending on the protein variant (43, 61, and 159 for 107 (G), 107 (K) and 107 (L), respectively), we will always name such mutation as M10V in reference to human CDKL5 wild type coordinates. As visible in Fig. 4c, such mutations abolished the synthesis of the truncated fragment in 107 (G) M10V and 107 (K) M10V producing strains. In the case of 107 (L) M10V, a lower molecular weight fragment at the level of the untagged protein was visible in anti-CDKL5 and anti-FLAG Western blots, but the intensity was low and comparable with all the other truncated fragments in the same lane. This suggests that such a product is unlikely to be caused by the internal translation initiation. An anti-FLAG Western blot specifically comparing the production profiles of pBCD2-107 (L) and pBCD2-107 (L) M10V strains proved this point (Additional file 1: Fig. S3). This result demonstrates that the addition of the globular Sumo domain at the N-terminal extremity of flCDKL5 did not resolve its fragmentation and that such fragmentation is unlikely to be due to proteolysis. Nevertheless, the 107 (L) M10V variant guaranteed better preservation of the N-terminal 6xHis tag than the 107 (G) and 107 (K) proteins (Fig. 4c, right panel). This outcome may be since in the latter variants the 6xHis tag is directly fused to extremely flexible regions (i.e. the C-terminal intrinsically disordered region in 107 (G) and the flexible N-terminal TATκ in 107(K), Fig. 4a).

As the last proof of an internal start occurring in flCDKL5 mRNA, we wanted to obtain the N-terminal amino acid sequence of the fragmented protein by using Edman sequencing. Since flCDKL5 is a difficult-to-purify protein, we tried to isolate such a protein fragment from a preparation of the CDKL5 catalytic domain only. We decided to produce this shortened version of CDKL5 in *Escherichia coli* rather than *P. haloplanktis* TAC125 given that previous reports demonstrate that this cell factory can be effectively used to isolate the CDKL5 catalytic domain [21]. To do so, we generated a short ORF encoding CDKL5(1-352) with a C-terminal His tag, performing a PCR on the 107 (L) encoding gene. Such construct was then expressed in *E. coli* BL21(DE3) cells using a pET system and the recombinant protein was isolated with an IMAC. As expected, the truncated fragment was co-purified and its N-terminal sequence was MNXF according to Edman degradation, where X is an undetermined amino acid. This indetermination was due to the little quantity of the protein fragment that could be recovered, but such a sequence is only compatible with a start in the M10 position in the CDKL5 protein, as expected. Hence, our results indicate that an internal translation start occurs when our engineered CDKL5 construct is expressed in bacteria which is typical of some eukaryotic cDNAs expressed in prokaryotes [42].

Based on this body of work, we selected pBCD2-107 (L) M10V as the best construct we tested for flCDKL5 expression. pBCD2 was preferred over pBCD1 because the latter only produced a truncated form of flCDKL5 (Fig. 4b). Moreover, pBCD2 was better than pBCD3 because the former guaranteed a higher accumulation of the full-length protein, as visible by anti-His Western blot (Fig. 4a, right panel) and confirmed by the pGFP results (Additional file 1: Fig. S2a). Furthermore, the pBCD2 configuration guaranteed increased stability of the flCDKL5 mRNA in comparison with the monocistronic p79C plasmid (2.73 ± 0.1 fold change), as demonstrated by qRT-PCR measurements that are in agreement with previous studies from other authors [43]. The 107 (L) variant was chosen because of the Sumo protective role on the N-terminal 6xHis tag (Fig. 4c, right panel). Finally, the M10V mutation was kept because it provided a solution to avoid the internal translational start. The presence of two different affinity tags at the two extremities of the protein constitutes a further advantage in sight of the future purification of flCDKL5. Double tagging has indeed been successfully used for the purification of both multidomain and intrinsically disordered proteins that are prone to fragmentation [19].

Overall, the multiparametric approach used to optimize flCDKL5 in *P. haloplanktis* TAC125 proved that the bicistronic strategy was pivotal to increase the yield of intact flCDKL5 from virtually absent to an amount detectable by Western blot. Nevertheless, BCDs were not sufficient to overcome an internal translational start, suggesting that the translation from the first and the internal ATGs are not in competition. This datum is corroborated by the fact that the abolition of translation from either the first ATG (pBCD2-107 (G) STOP in Fig. 4b) or the internal ATG (M10V mutants in Fig 4b) did not increase the production levels of the truncated and intact proteins, respectively, which is in agreement with a previous study [44]. Our hypothesis of testing multiple N- and C-terminal fusions with affinity tags proved to be valid because it allowed for better discrimination of flCDKL5 fragments during protein synthesis and proved that a bulky domain like Sumo can increase the preservation of the N-terminal 6xHis tag. This approach should be extended to the flCDKL5 C-terminal IDR in the pursuit of increased stabilization. Even if the main truncated fragment of flCDKL5 during recombinant expression in *P. haloplanktis* TAC125 was due to a translational issue, our Western blots highlight the existence of other numerous low molecular weight fragments though considerably less abundant. This pattern may be ascribable to the fragility of the flCDKL5 C-terminal tail and the fusion with a terminal globular domain could lessen its impact.

### Application of high-copy number plasmids for the expression of flCDKL5 and evaluation of the kinase activity

Very recently a library of replication origins with variable PCNs has been established for plasmid maintenance in *P. haloplanktis* TAC125 (Calvanese M. et al., manuscript in preparation). After we had optimized the flCDKL5 expression cassette, we moved it into a high copy number backbone named pB40 with an average PCN of ~ 100. The comparison of the expression profiles of the Antarctic cells bearing the original low copy number monocistronic plasmid p79C-107 (G) with the ones hosting the high copy number bicistronic plasmid pB40-BCD2-107 (L) M10V revealed a strong difference.

**Fig. 5:**
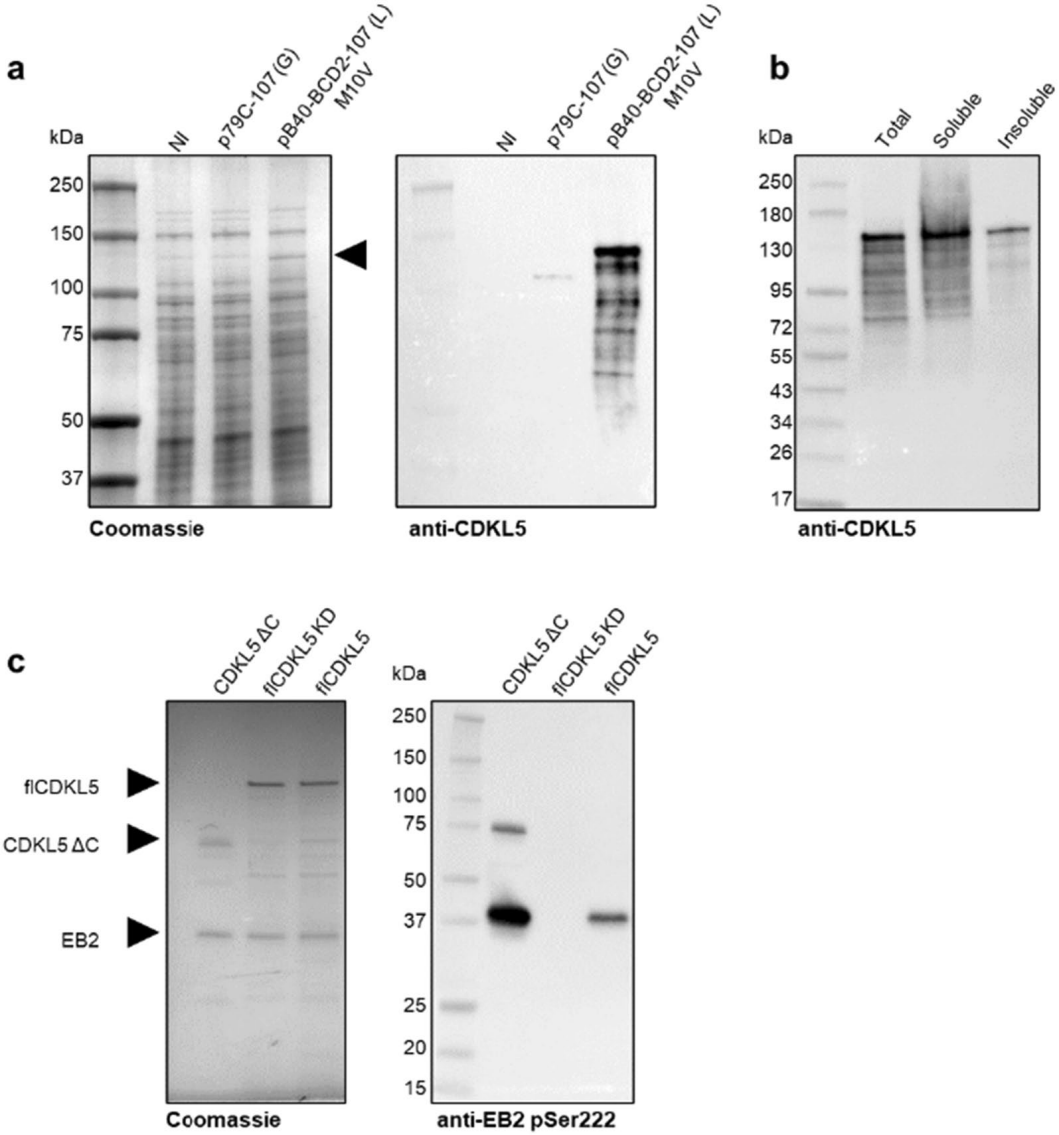
flCDKL5 production with a high copy number bicistronic plasmid.** a** Comparison of flCDKL5 expression levels in *P. haloplanktis* TAC125 recombinant strains harboring either a low copy number monoistronic plasmid (p79C-107 (G)) or an optimized high copy number bicistronic plasmid (pB40-BCD2-107 (L) M10V) using Coomassie staining after SDS-PAGE (left panel) and anti-CDKL5 Western blot (right panel). The arrow indicates flCDKL5 produced with the pB40-BCD2-107 (L) M10V plasmid. NI, not induced strain. **b** Solubility analysis of 107 (L) M10V protein after cellular lysis in native conditions. Total cellular extracts and soluble and insoluble fractions achieved after centrifugation were resolved via SDS-PAGE and the target protein was detected with an anti-CDKL5 Western blot. **c** Catalytic activity of enriched 107 (L) M10V on pure EB2. After chromatographic enrichment, either 107 (L) M10V (flCDKL5), or a catalytically inactive variant (flCDKL5 KD) was incubated with EB2 and MgATP and then assayed for catalytic activity. A commercial preparation of GST-CDKL5(1–498) from insect cells (CDKL5 ΔC) was used as a positive control. The total amount of the full-length enzyme and the substrate were detected by Coomassie staining (left panel, highlighted by arrows), while phosphorylated EB2 was observed with anti-EB2 pSer222 antibody (right panel)

While the protein achieved from the original construct was barely detectable by anti-CDKL5 Western blot, the optimized plasmid guaranteed a considerable higher accumulation of the target protein that was visible both by Coomassie staining after SDS-PAGE and by Western blot (Fig. 5a, left and right panels, respectively). Furthermore, when chemical-enzymatic lysis and fractionation of crude extracts were carried out in native conditions (see Materials and Methods for the details), most flCDKL5 appeared to be soluble (Fig. 5b). After a chromatographic enrichment with an anti-Flag resin, the recombinant kinase was tested for its catalytic activity by using an in vitro kinase assay with EB2 as a protein substrate. Briefly, 100 nM enzyme was incubated with 200 nM substrate in the presence of MgATP at 30 °C for 30 min. After protein inactivation with Laemmli buffer at 70 °C, the CDKL5-mediated phosphorylation of EB2 S222 was detected via Western blot with a specific antibody [9]. Remarkably, recombinant flCDKL5 enriched from *P. haloplanktis* TAC125 showed enzymatic activity on EB2, though it was less active than a commercial preparation of the CDKL5 catalytic domain from insect cells (CDKL5 ΔC, Fig. 5c). On the other hand, a catalytically inactive variant, flCDKL5 KD, did not phosphorylate EB2, as expected (Fig. 5c). The discrepancy between flCDKL5 and CDKL5 ΔC activities may be ascribable to more factors. First, the flCDKL5 C-terminal region is known to play an inhibitory effect on the enzymatic activity [7, 45, 46]. Second, the protein produced in the eukaryotic system may harbor higher phosphorylation levels that cannot be reached in a bacterial system. These two phenomena need to be dissected in the future by using CDKL5 ΔC purified from bacteria and flCDKL5 obtained from a eukaryotic system as controls, which is currently unavailable from commercial sources.

Besides these limitations, this work demonstrated that an increased gene dosage was needed to have satisfying flCDKL5 accumulation in *P. haloplanktis* TAC125 at 15 °C, a prerequisite that allowed the recovery of enough protein to assess its activity *in vitro*. To the best of our knowledge, no other bacterial platform has guaranteed to obtain flCDKL5 in native conditions as an active enzyme so far.

### Use of TCD plasmids to measure the impact of CDKL5 pathogenic mutations

**Fig. 6:**
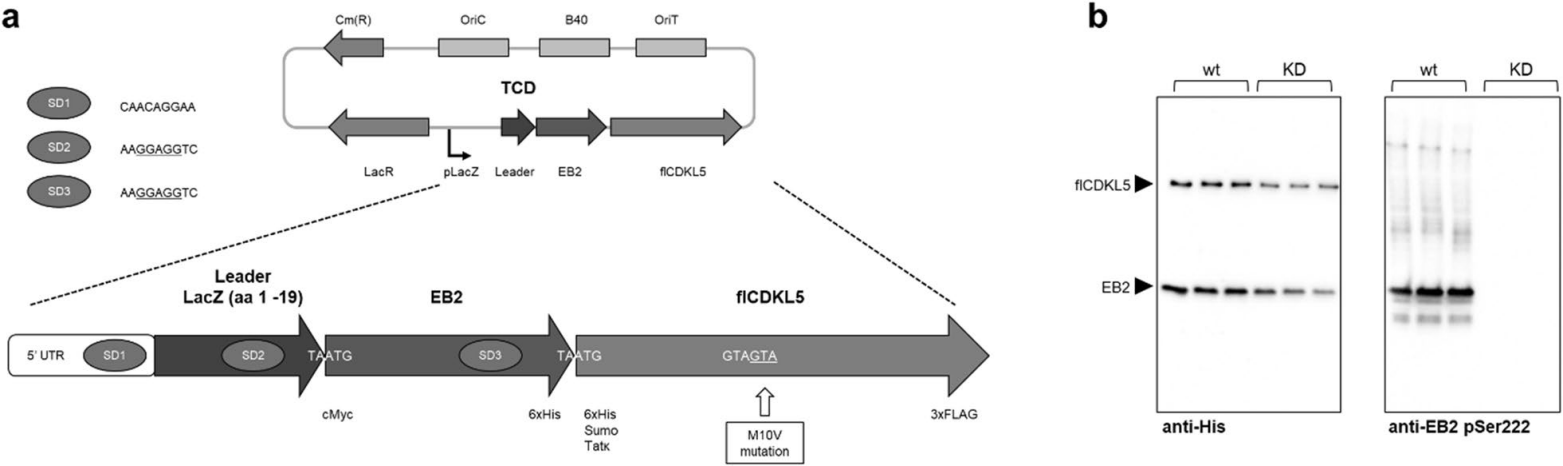
flCDKL5 and EB2 expression with a tricistronic plasmid.** a** Scheme of the TCD plasmid for the co-expression of flCDKL5 and EB2. The overall backbone was based on the optimized bicistronic pB40-BCD2 plasmid. Hence, it harbored a high copy number replication origin (B40); the selected Leader ORF and the SD1 are derived from LacZ; the SD2 and SD3 upstream of EB2 and flCDKL5 cistrons, respectively, harbor nucleotides perfectly complementary to 16S rRNA (underlined sequences in the inset). flCDKL5 was produced as 107 (L) M10V with the indicated tags and site-specific mutation, while EB2 was synthesized with an N-terminal cMyc and a C-terminal 6xHis tags. The translational coupling of the three cistrons was achieved through the overlap of a stop and a start codon (TAATG). **b** Co-expression of EB2 and either catalytically active flCDKL5 wt or inactive flCDKL5 KD. After recombinant expression, *P. haloplanktis* TAC125 recombinant crude lysates were analyzed via Western blot with an anti-His antibody to detect total proteins (left panel) and with an anti-EB2 pSer222 to detect specifically phosphorylated EB2 (right panel). Each assay was performed with biological triplicates (independent cultures) to have a qualitative idea of the robustness of the *in cellulo* assay. The two arrows serve to distinguish the flCDKL5 specific signal from the EB2 one

As the last proof of concept study, we planned to co-express flCDKL5 with one of its substrates to demonstrate that this prokaryotic platform can be used to validate CDKL5 substrates and model CDD mutations. The plasmid configuration optimized with the BCD2 design was converted into a tricistronic plasmid for the co-expression of EB2 and flCDKL5. As schematized in Fig. 6a, the TCD plasmid triggered the production of a polycistronic mRNA encoding the LacZ leader peptide, EB2 and flCDKL5.

To test whether this system can be used to reliably measure flCDKL5 catalytic activity through an in cellulo kinase assay, EB2 was co-expressed either with flCDKL5 or flCDKL5 KD, a catalytically inactive variant. As visible in Fig. 6b (left panel), the synthesis of EB2 and flCDKL5 could be contextually detected with an anti-His antibody and was consistent through biological triplicates. Furthermore, specific CDKL5-mediated phosphorylation of EB2 could be detected only when active flCDKL5 was produced. This result confirms that this assay can be used to undoubtedly measure CDKL5 specific activity.

**Fig. 7:**
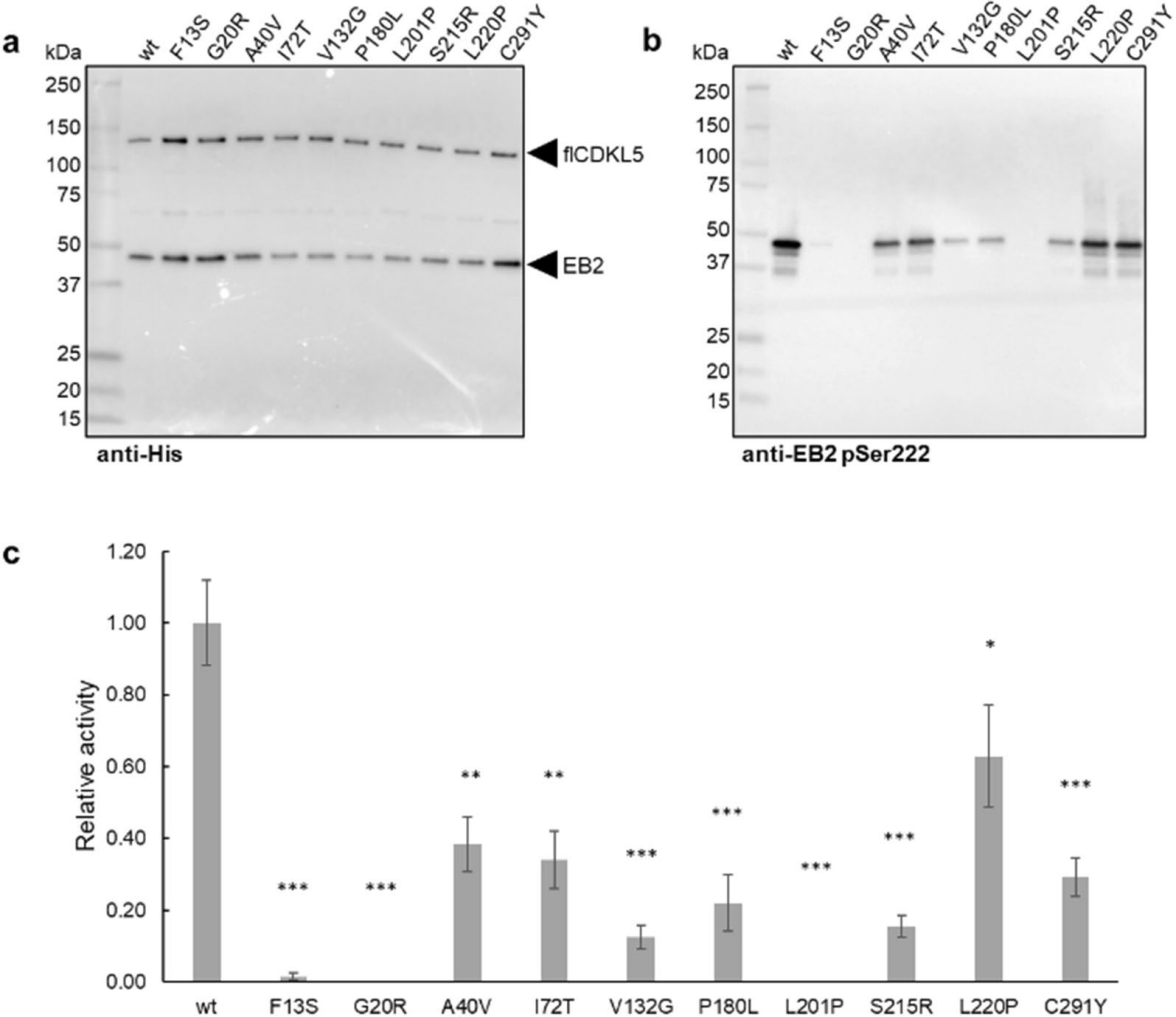
Effect of pathogenic CDKL5 mutations on flCDKL5 activity. **a** flCDKL5 variants and EB2 were co-expressed in *P. haloplanktis* TAC125 using TCD plasmids. After cellular lysis, flCDKL5 mutants and EB2 were detected in each crude lysate using an anti-His antibody after Western blot, as indicated by the two arrows. **b** Phosphorylated EB2 was detected with an anti-EB2 pSer222 antibody. **c** EB2 phosphorylation levels were normalized for total EB2 and flCDKL5. The results are reported as the mean of technical triplicates and the error bars represent standard deviations. **a** and **b** show Western blots representative of one experiment. * p < 0.05, ** p < 0.01, *** p < 0.001. Note: every CDKL5 construct (also the wt) harbors an M10V mutation besides the one indicated in the figure. Mutation coordinates are referred to the human CDKL5 isoform 1 (#O76039, Uniprot)

Most of the patients affected by CDKL5 Deficiency Disorder (CDD) suffer from refractory epilepsy, hypotonia, intellectual and motor disabilities, and visual impairments [3]. However, the severity of these symptoms is variable, and clear genotype-phonotype correlations are scarce. For this reason, we wanted to test if our co-expression assay can be a valid tool to model CDD CDKL5 variants at the molecular level. To do so, 10 pathogenic flCDKL5 missense variants were co-expressed with EB2 using TCD plasmids, and EB2 phosphorylation levels were quantified. All flCDKL5 variants were hypoactive and the levels of EB2 cross-phosphorylation were variable, indicating a different impact of each mutation on flCDKL5 functionality (Fig. 7). This kind of study can be extended in the future to other known CDKL5 substrates to have a deeper understanding of CDD pathogenesis.

## Conclusions

Our multivariate approach to optimize the production of flCDKL5 in *P. haloplanktis* TAC125 at 15 °C led to the accumulation of the target protein mainly as an intact, soluble, and active form, making feasible further developments for its purification and formulation. Furthermore, the co-expression assay with EB2 demonstrated two main points: (1) flCDKL5 is active when properly produced in bacteria; and (2) CDD missense variants can be modeled and discretized based on their hypoactivity. Such an approach will be useful in the future to support the reconstruction of CDKL5-mediated networks and to characterize the molecular effect of CDD mutations.

In addition, our results further support the more general potentialities of exploitation of the Antarctic bacterium to produce “intractable” proteins such as those endowed with a folded N-terminal domain and a long disordered tail, a common trait in the human kinome. Notably, all RCK kinases that are involved in cilia biology share such property, and our work demonstrate that *P. haloplanktis* TAC125 could be a suitable host to test their expression and purification for further characterization.

## Methods

### Bacterial strains and culture conditions

*E. coli* TOP10 was used for cloning purposes, while *E. coli* S17-1(λpir) was employed in intergeneric conjugations as a donor strain for *P. haloplanktis* TAC125 transformations. *E. coli* BL21(DE3) was used for the recombinant production of the catalytic domain of human CDKL5 and for the synthesis of human EB2.

The *P. haloplanktis* TAC125 strain that was used for the expression of flCDKL5 alone is KrPl LacY^+^ which lacks its endogenous plasmid pMtBL and the *lon* gene, and constitutively expresses the *E. coli* lactose permease [31]. For the double production of EB2 and flCDKL5, the KrPl strain was used, instead [31]. The induction of recombinant expression was performed at 1 OD with either 10 mM D-galactose or 5 mM IPTG for pMAV and p79C-derived plasmids, respectively. If not differently specified, the recombinant expression was protracted for 4 h.

The *E. coli* strains were grown in Lysogen broth (LB, 10 g/L bacto-tryptone, 5 g/L yeast extract, 10 g/L NaCl) at 37 °C with 200 rpm agitation. The BL21(DE3) recombinant strains were induced in mid-exponential phase with 0.1 mM IPTG at 15 °C for 16 h. *P. haloplanktis* TAC125 was grown in a rich medium (16 g/L bacto-tryptone, 16 g/L yeast extract, 10 g/L NaCl) during the preinocula development, and in a synthetic medium named GG for recombinant expression [30]. In both cases, the bacterial cultures were carried out at 15 °C with 200 rpm agitation.

For the selection of *E. coli* recombinant strains, either 100 µg/mL ampicillin (for pMAV derived plasmids) or 34 µg/mL chloramphenicol (for p79C derived plasmids) or 50 µg/mL kanamycin (for pET40-b derivatives) was used, depending on the selection marker. In the case of recombinant *P. haloplanktis* TAC125, chloramphenicol was used at 12.5 µg/mL in solid media and 25 µg/mL in liquid media, while ampicillin was always used at 100 µg/mL concentration.

### Plasmids construction

Three genes encoding different flCDKL5 variants were first cloned into two monocistronic plasmids. Then, to test flCDKL5 expression in bicistronic configurations with different N- and C-terminal tags, BCDs were developed. To do so, two flCDKL5 genes were cloned into Bicositronic Entry Clone plasmids (BECs) and then short DNA fragments harboring different Leader ORFs, Shine Dalgarno sequences and N-terminal tags were inserted between the pLacZ promoter and the gene of interest (GOI). The same procedure was followed for the preparation of the pGFP-based vectors. The list of the primers used in this work is reported in Additional file 1: Table S2.

#### Monocistronic designs

For the recombinant expression of flCDKL5 with monocistronic plasmids, pMAV [30], and p79C [31] vectors were used. The genes encoding flCDKL5 variants named 107 (B), 107 (G) and 107 (H) were synthesized by an external company and cloned into pMAV by using NdeI/EcoRI double digestion. The transfer of the genes of interest from pMAV into p79C was achieved with the use of NdeI and SacI allowing the isolation of the recombinant genes together with the transcriptional terminator [31]. The sequences of the three genes are reported in the Additional file 1. 107 (B) and 107 (G) have a different codon composition, but they encode the same protein with an N-terminal TATκ peptide and C-terminal tandem 6xHis and 3xFLAG tags. 107 (H) differs from the other two variants for the lack of a His tag.

#### Entry clone designs

For the design of bicistronic plasmids (BCDs), Entry Clone plasmids (BECs) were prepared first. To do so a parental plasmid, p79BsC, was generated by cloning a synthetic fragment encompassing the LacR and pLacZ sequences of p79C into the pUCC backbone [47] using SphI/PstI double digestion (Additional file 1: Fig. 4, left plasmid). The aim of this step was the introduction of a BsaI site downstream of the pLacZ promoter.

The three BEC plasmids that were then generated shared the common feature of harboring two divergent BsaI sites upstream of the GOI, so as to allow the scarless cloning of DNA fragments between the pLacZ promoter and the GOI with the Golden Gate technology [48]. pBEC-pGFP was generated by isolating the pGFP gene from p79C-pGFP [31] via PCR using the *pGFP_PstIBsaI_fw* and *pGFP_KpnI_rv* primers, and cloning it into p79BsC with PstI/KpnI digestion (Additional file 1: Fig. S4, upper branch).

pBEC-107 (G) was developed by cloning the 107 (G) gene as two fragments, 5′ 107 and 3′ 107 (G). The former is a synthetic DNA harboring the first half of the flCDKL5 gene with left PstI and BsaI sites and a right HindIII site. 3′ 107 (G)—the second half of the gene—was extracted from p79C-107 (G) using HindIII/SacI double digestion. The two fragments were cloned between PstI and SacI into p79BsC (Additional file 1: Fig. S4, middle branch).

pBEC-107 (H) was generated in the same way as pBEC-107 (G) with the only difference that 3′ 107 (H) was used in place of 3′ 107 (G). Such fragment was obtained from p79C-107 (H) using HindIII/SacI digestion and was cloned together with 5′ 107 into p79BsC (Additional file 1: Fig. S4, bottom branch).

#### Bicistronic designs

To generate the various pBCD plasmids described in this work, different DNA fragments, named Strings A-F were cloned into the BEC plasmids. As depicted in Additional file 1: Fig. S5a, each String has two divergent BsaI sites at the extremities to allow Golden Gate cloning. Furthermore, every String harbors the 5’UTR of p79C (+ 1 in Additional file 1: Fig. S5a indicates the transcription start) and different combinations of SD1, SD2, Leader ORF and possibly N-terminal tags to be fused to the GOI. The various combinations of BEC plasmids and Strings generated the pBCDs described in this work (Additional file 1: Fig. S5b). Finally, to generate pBCD2-H6-TATκ-pGFP, a PCR on pBCD2-107 (K) was performed with *pBCD_SphI_fw* and *H6-TATk_BsaI_rv* primers to isolate a DNA fragment harboring the H6-Tatκ encoding sequence to be cloned into pBEC-pGFP upstream of the pGFP gene.

Mutagenesis reactions into flCDKL5 encoding genes were carried out with the QuikChange II XL Site-Directed Mutagenesis kit (Agilent). In particular, the elimination of the first ATG was achieved using *M1I_G2stop_fw* and *M1I_G2stop_rv* primers; M10V mutations were performed with *M10V_fw* and *M10V_rv* primers; Kinase Dead mutants were obtained using *Mut_KK_fw* and *Mut_KK_rv*.

#### High copy number bicistronic plasmids

The low copy number OriR of the recombinant plasmids was replaced by the high copy number B40 replication origin using AscI/NotI double digestion.

#### Tricistronic designs

To co-express the LacZ Leader peptide, EB2 and flCDKL5, a similar strategy as for the BCD plasmids was applied. First, pB40-BCD2-107 (L) M10V was converted into an Entry Clone plasmid (pB40-BEC-107 (L) M10V). To do so, a PCR fragment encompassing the first half of the 107 (L) M10V gene was amplified with *CDKL5_L_PstIBsaI_fw* and *CDKL5_NheI_rv* primers to introduce PstI and BsaI sites upstream of the 6xHis-Sumo tag encoding sequence. Then, such DNA fragment was cloned into pB40-BEC-107 (H) using PstI/NheI double digestion so as to replace the 5’ half of the flCDKL5 gene and generate pB40-BEC-107 (L) M10V (Additional file 1: Fig. S6a). Finally, the BEC plasmid was converted into the tricistronic plasmid (TCD) by cloning a DNA fragment harboring the 5’UTR, the LacZ (aa 1–19) encoding sequence, and the cmyc-EB2-6xHis gene between the two BsaI sites (Additional file 1: Fig. S6b).

For the development of TCD plasmids expressing CDD mutants of flCDKL5, TCD was subjected to mutagenesis with the QuikChange II XL Site-Directed Mutagenesis kit (Agilent) with the primers listed in Additional file 1: Table S2.

### Measurement of average plasmid copy number (PCN)

The quantification of p79C-107(B) in *P. haloplanktis* TAC125 was carried out as previously reported [35]. Briefly, total DNA was extracted from 1 OD of bacterial cells using the E.Z.N.A. Bacterial DNA kit (Omega Bio-Tek Inc). Then, five serial dilutions of total DNA were employed as substrates in qPCR reactions with either *Prom7_fw* and *Prom7_rv* primers to detect the genomic DNA, or *CDKL5_fw* and *CDKL5_rv* to quantify the plasmid. Hence, the PCN values were calculated as follows: PCN = E_c_^Ctc^ / E_p_^Ctp^, where Ec and Ep are the efficiencies obtained from the standard curves of the amplification of the chromosomal and plasmid genes, respectively, and Ctc and Ctp are the threshold cycles for the two amplicons (chromosomal and plasmid genes) in each sample.

### Quantification of flCDKL5 mRNA

Total RNA was isolated using the Direct-zol RNA Kit (Zymo Research, Irvine, CA, USA) adopting the manufacturer’s instructions, followed by treatment with RNAse-free DNase I (Roche, Mannheim, Germany) to avoid genomic DNA contamination. Total RNA was reverse transcribed using SuperScript IV (Invitrogen, Carlsbad, CA, USA) according to the recommended protocol using *PSHA_RS01090_rv* and *CDKL5_rv* primers. 1 μL cDNA from each sample was used as the template for quantitative real-time PCR by using 1X PowerUp SYBR Green Master Mix (Applied Biosystems, Foster City, CA, USA) in the presence of 400 nM of specific primers (*PSHA_RS01090 _fw*, *PSHA_RS01090 _rv*; *CDKL5_fw*, *CDKL5_rv*). The reactions were run by a StepOne Real-time PCR System (Applied Biosystems, Foster City, CA, USA) and three independent sets of experiments were performed.

The thermal cycling protocol was set up as follows: UDG activation for 2 min at 50 °C; initial denaturation for 10 min at 95 °C; 40 cycles of denaturation for 15 s at 95 °C alternated with annealing/extension steps for 1 min at 60 °C. At the end of each reaction, melting curves were performed to verify the presence of a specific and unique amplification product. The housekeeping gene PSHA_RS01090 was chosen as the normalizer for variations of mRNA amounts, cDNA synthesis efficiency and plasmid DNA contamination. The expression level of the CDKL5 gene was assayed for up-regulation in the experimental samples (induction of expression) in comparison to the calibrator sample (noninduced cells, NI). The relative quantification of mRNA was expressed as fold-change and was calculated through the standard curve method (Pfaffl method):

Gene expression ratio = (E_target_) ^ΔCt^_target_^(control−sample)^ /(E_housekeeping_) ^ΔCt^_housekeeping_.^(control−sample)^ [49]

### Measurement of pGFP fluorescence

After recombinant production, the equivalent of 1 OD cells was harvested and washed with PBS. Then, each sample was diluted with the same buffer to achieve the best signal-to-noise ratio in the fluorescence measurements that were carried out with a JASCO FP-750 spectrofluorometer at 25 °C (excitation at 488 nm and emission at 509 nm).

### Analysis of the production of flCDKL5 and EB2 proteins

To analyze total protein productions, 1 OD pellets were solubilized in Laemmli Sample buffer and heated at 90 °C for 20 min. Then, total cellular extracts were resolved by SDS-PAGE and analyzed either by Coomassie staining or Western blot. For the solubility analysis, cells were lysed in 20 mM sodium phosphate buffer pH 7.0, supplemented with 0.5 M NaCl, 10% Glycerol, 0.1% Triton X-100, 20 U/mL DNAse I, 0.1 mg/mL lysozyme, 1 mM DTT and a protease inhibitor cocktail. Then, the soluble and insoluble fractions were segregated by centrifugation. Finally, the insoluble fraction was resuspended with lysis buffer in the same volume as the soluble fraction. 10 µg of soluble extract were analyzed by SDS-PAGE and the same volume of insoluble fraction was used as a control of solubility.

After SDS-PAGE runs, proteins were electroblotted to PVDF membranes using a semidry system. After the incubation with specific antibodies, the chemiluminescent signals were developed with the ECL method.

To detect flCDKL5, the membrane was blocked with PBS, 0.05% Triton X-100, 5% w/v milk for one hour. Then, CDKL5 (D-12): sc-376314 antibody (Santa Cruz Biotechnology) was diluted 1:1000 in the same buffer. After one hour of incubation at room temperature with the primary antibody, the membrane was washed with PBS, 0.05% v/v Triton X-100 three times (5 min each) and incubated with an anti-mouse antibody diluted 1:10,000 in PBS, 0.05% v/v Triton X-100, 5% w/v milk for one hour. Then, the membrane was washed again with PBS, 0.05% v/v Triton X-100 three times and the secondary antibody was detected using the ECL method.

For anti-FLAG Western blots, the membrane was blocked with PBS, 0.2% Tween 20, 5% w/v milk, for one hour. Then, Monoclonal ANTI-FLAG M2, Clone M2 (F1804, Sigma) was diluted 1:1000 in the same buffer. After overnight incubation at 4 °C with the primary antibody, the membrane was washed with PBS, 0.2% Tween 20 three times and incubated with an anti-mouse antibody diluted 1:5000 in PBS, 0.2% Tween 20, 5% w/v milk for one hour at room temperature. Then, the membrane was washed again with PBS, 0.2% Tween 20 three times and the secondary antibody was detected using the ECL method.

In the case of anti-His Western blots, the membrane was blocked with PBS, 5% w/v milk for one hour. Then, Monoclonal Anti-polyHistidine-Peroxidase clone HIS-1 antibody (A7058, Merck) was diluted 1:2000 in PBS, 0.05% Tween 20, 5% w/v milk. After one hour of incubation at room temperature with the antibody, the membrane was washed with PBS, 0.05% Tween 20 three times and it was developed.

To measure EB2 phosphorylation of Ser222, the membrane was blocked with TBST, 5% w/v milk for one hour. Then, anti-EB2 pS222 antibody (00117739, Covalab) was diluted 1:4000 in the same buffer. After overnight incubation at 4 °C with the primary antibody, the membrane was washed with TBST three times and incubated with an anti-rabbit antibody diluted 1:2000 in TBST and 5% w/v milk for one hour at room temperature. Then, the membrane was washed again with TBST three times and was developed.

Parallel Coomassie stained polyacrylamide gels were always used to ascertain that complex samples (i.e. total and soluble lysates) were correctly balanced in Western blot analyses.

### Preparation of samples for N-terminal sequencing

To produce the catalytic domain of CDKL5 in *E. coli*, the primers named *PhSumoCDKL5_NdeI_fw* and *PhCDKL5dC_XhoI_rv* were used in a PCR on pB40-BCD2-107 (L). This gene encoding a Sumo-tagged version of CDKL5(1–352) was cloned into the pET40-b vector in frame with a C-terminal 8xHis tag using NdeI/XhoI double digestion. After recombinant expression in *E. coli* BL21(DE3), the recombinant cells were resuspended in 50 mM TrisHCl pH 8.0, 0.5 M NaCl, 20 mM imidazole and lysed by sonication in the presence of a protease inhibitor cocktail. After centrifugation (14,000 *g*, 4 °C, 60 min), the soluble fraction was loaded onto a HisTrap of 1 mL (Cytiva) and both the full-length protein, and its N-terminally truncated fragment were collected with a linear gradient of imidazole. A sample containing approximately 30 µg of the intact catalytic domain and 6 µg of the N-terminally truncated fragment were loaded onto SDS-PAGE and then electroblotted onto a PVDF membrane using 10 mM CAPS, 10% methanol pH 11.0 as the transfer buffer. The protein bands were made visible by Ponceau S staining and submitted to Edman sequencing at the Institute of Biosciences and Bioresources (CNR, Naples).

### Enrichment of 107 (L) M10V from *P. haloplanktis* TAC125 lysate

Recombinant *P. haloplanktis* TAC125 was lysed with a chemical-enzymatic method. Briefly, the cell paste was resuspended in 20 mM sodium phosphate buffer pH 7.0, supplemented with 0.5 M NaCl, 10% glycerol, 0.1% Triton X-100, 20 U/mL DNAse I, 0.1 mg/mL lysozyme, 1 mM DTT, and a protease inhibitor cocktail to reach a final concentration of 14 OD/mL. After incubation at 4 °C for 20 min, the suspension was centrifugated (14,000 *g* for 45 min at 4 °C) to separate the soluble fraction from the cellular debris. Then, the soluble lysate was incubated with 0.15 mL of ANTI-FLAG M2 Affinity gel (Millipore) at 4 °C for 4 h and a gravity flow column chromatography was performed. The resin was washed with lysis buffer containing 1% Triton X-100, while the elution was performed using in 0.5 mL of the same buffer containing 175 µM 3xFLAG peptide.

### EB2 purification

A codon optimized EB2 gene was synthesized by an external company and cloned into the pET40-b with NdeI/BamHI double digestion. The resulting gene encodes human EB2 with an N-terminal 6xHis tag (see the Additional file 1: Information for the nucleotide sequence). After recombinant expression, *E. coli* BL21(DE3) recombinant cells were lysed by sonication in 50 mM TrisHCl pH 8.0, 0.5 M NaCl, 5% glycerol, 20 mM imidazole supplemented with a protease inhibitor cocktail. The soluble fraction was recovered after a centrifugation (14,000 *g* for 45 min at 4 °C) and loaded onto a HisTrap of 1 mL (Cytiva). The target protein was eluted with 250 mM imidazole and loaded onto a a Hiload 16/600 Superdex 200 pg using 50 mM TrisHCl pH 8.0, 0.18 M NaCl as a running buffer for a final polishing step. The final preparation was stored at – 80 °C in 40 mM TrisHCl pH 8.0, 0.15 M NaCl, 1 mM DTT, 15% glycerol at 1.8 mg/mL protein concentration.

### In vitro kinase assay

EB2 phosphorylation assays with enriched CDKL5 proteins were carried out using 200 nM EB2 and 100 nM of enzyme in 30 μL of 20 mM TrisHCl pH 7.7, 0.5 M NaCl, 10 mM MgCl_2_, 1 mM DTT, 0.7 mM ATP, complete protease inhibitor cocktail (Roche) and Halt phosphatase inhibitor cocktail (Thermo Fisher Scientific). The reactions were stopped after 30 min with 10 μL Laemmli Sample buffer 4 × and denatured at 70 °C for 20 min. 10 μL of each reaction were analyzed via either SDS-PAGE (for total CDKL5 and EB2 detection) or anti-EB2 pSer222 Western blot (for phosphorylated EB2 detection). As a negative control, a reaction was set up with flCDKL5 KD, a catalytically inactive CDKL5 variant. As a positive control, a reaction with commercial GST-CDKL5(1–498) (ab131695, abcam) was performed.

### Statistics and reproducibility of results

The Data from the *in cellulo* kinase assays were statistically validated using the t-Student test comparing the mean measurements of experimental and control samples, both carried out as technical triplicates. The significance of differences between mean values was calculated using a two-tailed Student’s t-test. A p value of < 0.05 was considered significant.

## Supplementary Information

**Additional file 1: Table S1.** Characteristics of BCD constructs for pGFP expression. **Table S2.** List of primers used in this work. **Fig. S1.** Average plasmid copy number (PCN) of pP79-107 (B). **Fig. S2.** Ranking of bicistronic designs (BCDs) with a fluorescent reporter. **Fig. S3.** flCDKL5 production profiles with pBCD-107 (L) plasmids. **Fig. S4.** Development of Bicistronic Entry Clones. **Fig. S5.** Development of Bicistronic Designs. **Fig. S6.** Development of Tricistronic Designs.

## Data Availability

The sequences of the original flCDKL5 encoding genes (107 (B), 107 (G), 107 (H)) cloned into pUC18 have been deposited in GenBank with the accession codes ON605205, ON605206 and ON605207, respectively. The synthetic plasmid pMK-T-H6EB2 from which the EB2 encoding gene was taken for expression in *E. coli* is available in GenBank with the accession code ON605208, while the sequence for EB2 co-expression with flCDKL5 was obtained from pMK-RQ-BCD_LacZ-cmyc-EB2-H6, whose accession code in GenBank is ON605209. All the other constructs described in this work were derived from such sequences and the ones described in refs. [30, 31]. Correspondence and material requests should be addressed to MLT (tutino@unina.it) and AC (andrea.colarusso@unina.it).
