# Supplementary material for: Active human full-length CDKL5 produced in the Antarctic bacterium Pseudoalteromonas haloplanktis TAC125
Source: Microb Cell Fact. 2022 Oct 14;21:211. doi: 10.1186/s12934-022-01939-6 (PMC9563788; doi:10.1186/s12934-022-01939-6)
Supplement: Supplementary file 1 — Additional file 1: Table S1. Characteristics of BCD constructs for pGFP expression. Table S2. List of primers used in this work. Fig. S1. Average plasmid copy number (PCN) of pP79-107 (B). Fig. S2. Ranking of bicistronic designs (BCDs) with a fluorescent reporter. Fig. S3. flCDKL5 production profiles with pBCD-107 (L) plasmids. Fig. S4. Development of Bicistronic Entry Clones. Fig. S5. Development of Bicistronic Designs. Fig. S6. Development of Tricistronic Designs. [file 12934_2022_1939_MOESM1_ESM.docx]

**ADDITIONAL MATERIAL**

**TO**

**Active human full-length CDKL5 produced in the Antarctic bacterium *Pseudoalteromonas haloplanktis* TAC125**

**Andrea Colarusso^1,2^, Concetta Lauro^1,2^, Marzia Calvanese^1^, Ermenegilda Parrilli^1^, Maria Luisa Tutino^1,*^**

^1^ Dept. of Chemical Sciences, “Federico II” University of Naples, Complesso Universitario Monte S. Angelo- via Cintia, 80126 Napoli Italia.

^2^ Istituto Nazionale Biostrutture e Biosistemi—I.N.B.B., Viale Medaglie d’Oro, 305-00136 Roma, Italy

* correspondence to [tutino@unina.it](mailto:tutino@unina.it)

- table S1- Characteristics of BCD constructs for pGFP expression.
- table S2- List of primers used in this work
- figure S1- Average plasmid copy number (PCN) of pP79-107 (B)
- figure S2- Ranking of bicistronic designs (BCDs) with a fluorescent reporter
- figure S3- flCDKL5 production profiles with pBCD-107 (L) plasmids
- figure S4- Development of Bicistronic Entry Clones
- figure S5- Development of Bicistronic Designs
- figure S6- Development of Tricistronic Designs
- Nucleotide sequences of flCDKL5 constructs cloned into pMAV and p79

**Table S1.** Characteristics of BCD constructs for pGFP expression.

| Name | Leader^a^ | SD1^b^ | SD2^c^ | N-tags^d^ |
| --- | --- | --- | --- | --- |
| p79C-pGFP | / | / | CAACAGGAA | / |
| pBCD1-pGFP | LacZ | CAACAGGAA | CAACAGGAA | / |
| pBCD2-pGFP | LacZ | CAACAGGAA | AAGGAGGTC | / |
| pBCD2-H6-TATκ -pGFP | LacZ | CAACAGGAA | CAACAGGAA | 6xHis-TATκ |
| pBCD3-H6-pGFP | TrpA | AAGGAGGTC | AAGGAGGTC | 6xHis |

^a^Leader peptide encoded upstream of pGFP. ^b^Shine Dalgarno sequence upstream of the Leader peptide encoding sequence. ^c^Shine Dalgarno sequence upstream of the pGFP gene. Nucleotides perfectly complementary to 16S rRNA are underlined. ^d^N- tags are referred to pGFP protein.

**Table S2.** List of primers used in this work.

| **Primer name** | **Sequence (5' - 3')** | **Purpose** |
| --- | --- | --- |
| pGFP_PstIBsaI_fw pGFP_KpnI_rv | AACTGCAGAAGGTCTCAATGGTATCTAAAGGTG TTCGGTACCTTATTTGTAAAGCTCGTC | Genesis of pBEC-pGFP |
| pBCD_SphI_fw H6-TATk_ BsaI_rv | TAAGCATGCACCATGATAACGATGAT TTGGTCTCACCATGCTGCCACCACCACCAGCA | Isolation of a String with 5' UTR, SD1, SD2, and Tatκ-H6 tag |
| M1I_G2stop_fw M1I_G2stop_rv | GAAAATAAGGAGGTCAAATAATTTGAGATGCGGCACAACCTGCAC GTGCAGGTTGTGCCGCATCTCAAATTATTTGACCTCCTTATTTTC | Elimination of of the first ATG in flCDKL5 encoding genes |
| M10V_fw M10V_rv | CCCTAACATTGGTAATGTAGTAAACAAATTCGAAATTCTTGGTG CACCAAGAATTTCGAATTTGTTTACTACATTACCAATGTTAGGG | M10V replacement in flCDKL5 encoding genes |
| Mut_KK_fw Mut_KK_rv | ACACACGAAATAGTAGCGATCAGGAGGTTCAAAGATTCTGAAGAG CTCTTCAGAATCTTTGAACCTCCTGATCGCTACTATTTCGTGTGT | KK42,43RR flCDKL5 Kinase Dead mutation |
| CDKL5_L_PstIBsaI_fw CDKL5_NheI_rv | AACTGCAGAAGGTCTCAATGGGTCACCATCAC TACTTCCTGGTTGGCTGCTAGCTTG | Genesis of pB40-BEC-107 (L) M159V |
| PhCDKL5_M10V_F13S_fw PhCDKL5_M10V_F13S_rv | GCAAAATCCCTAACATTGGTAATGTAGTAAACAAAAGCGAAATTCTTGGTGTAGT ACTACACCAAGAATTTCGCTTTTGTTTACTACATTACCAATGTTAGGGATTTTGC | M10V/F13S mutation in CDKL5 |
| PhCDKL5_G20R_fw PhCDKL5_G20R_rv | CGAAATTCTTGGTGTAGTACGTGAAGGTGCTTACGG CCGTAAGCACCTTCACGTACTACACCAAGAATTTCG | G20R mutation in CDKL5 |
| PhCDKL5_A40V_fw PhCDKL5_A40V_rv | CAAAGAGACACACGAAATAGTAGTGATCAAGAAGTTCAAAGATTCTG CAGAATCTTTGAACTTCTTGATCACTACTATTTCGTGTGTCTCTTTG | A40V mutation in CDKL5 |
| PhCDKL5_I72T_fw PhCDKL5_I72T_rv | AATGCTGCGTACACTAAAGCAAGAAAACACTGTTGAATTAAAAGAGG CCTCTTTTAATTCAACAGTGTTTTCTTGCTTTAGTGTACGCAGCATT | I72T mutation in CDKL5 |
| PhCDKL5_V132G_fw PhCDKL5_V132G_rv | GTGTCATAAAAACGACATAGGTCACAGGGACATTAAACCTG CAGGTTTAATGTCCCTGTGACCTATGTCGTTTTTATGACAC | V132G mutation in CDKL5 |
| PhCDKL5_P180L_fw PhCDKL5_P180L_rv | TCGTTGGTATCGCAGCCTTGAACTTCTACTAGGTG CACCTAGTAGAAGTTCAAGGCTGCGATACCAACGA | P180L mutation in CDKL5 |
| PhCDKL5_L201P_fw PhCDKL5_L201P_rv | CGTCAGAAAGTTCACCAGGGATACATCCTACTGAC GTCAGTAGGATGTATCCCTGGTGAACTTTCTGACG | L201P mutation in CDKL5 |
| PhCDKL5_L215R_fw PhCDKL5_L215R_rv | GCCTCTTTTCCCAGGTGAGAGAGAAATCGACCAACTTTTC GAAAAGTTGGTCGATTTCTCTCTCACCTGGGAAAAGAGGC | L215R mutation in CDKL5 |
| PhCDKL5_L220P_fw PhCDKL5_L220P_rv | GTGAGTCAGAAATCGACCAACCTTTCACAATTCAAAAGGTACT AGTACCTTTTGAATTGTGAAAGGTTGGTCGATTTCTGACTCAC | L220P mutation in CDKL5 |
| PhCDKL5_C291Y_fw PhCDKL5_C291Y_rv | ATCGCTACCTCACTGAACAGTATCTAAATCATCCGAC GTCGGATGATTTAGATACTGTTCAGTGAGGTAGCGAT | C291Y mutation in CDKL5 |
| CDKL5_fw CDKL5_rv | ACTCGTTGGTATCGCAGCCC TGGCCGTCAGAAAGTTCACCA | qPCR to quantify flCDKL5 mRNA and the PCN of p79C-107(B) |
| Prom7_fw  Prom7_rv | CCTTTATTCAGCGTGTTGGCGAGC  GTTATCAGGGTCGGGCGTATCGG | qPCR of PSHA_RS10135 gene |
| PHSA_R01090_fw PHSA_R01090_rv | CTAAAGACCAAATCCTTGACGCA GACCAGCTACCATACCAGCA | qPCR to quantify PHSA_R01090 mRNA |
| PhSumoCDKL5_NdeI_fw PhCDKL5dC_XhoI_rv | AAACATATGGGTTCGGACTCAGAAGTA AAAACTCGAGGCCAACAGAAAGATTCT | Cloning of the CDKL5 catalytic domain encoding gene into pET40b |

**
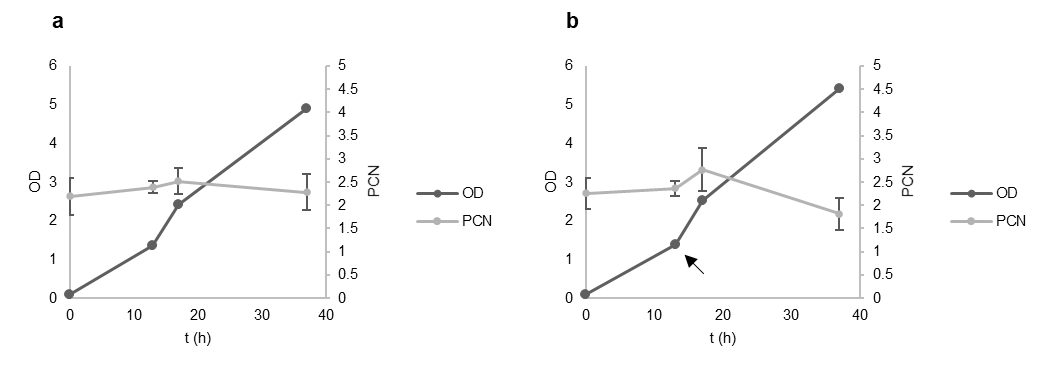
**

**Fig. S1** Average plasmid copy number (PCN) of pP79-107 (B) in *P. haloplanktis* TAC125. *P. haloplanktis* TAC125 pP79-107 (B) was grown at 15 °C either in absence of IPTG (**a**) or induced with 5 mM IPTG 13 h after the initial dilution (**b**). A defined quantity of cells was taken at increasing time points and the PCN was defined with qPCR. Error bars indicate standard deviations from triplicate assays. The arrow in (**b**) indicates the induction point.


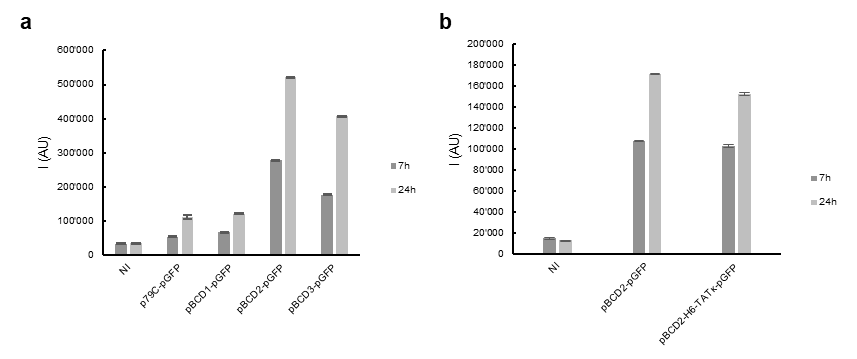


**Fig. S2** Ranking of bicistronic designs (BCDs) with a fluorescent reporter. **a** Comparison of bicistronic plasmids pBCD1, pBCD2 and pBCD3 with the monocistronic plasmid p79C, by using the fluorescent reporter pGFP. **b** Evaluation of the influence of the N-terminal H6-Tatκ tag on the production of pGFP in the BCD2 configuration. The results are reported as the mean of technical triplicates and the error bars represent standard deviations. AU, arbitrary units; NI, not induced.

**
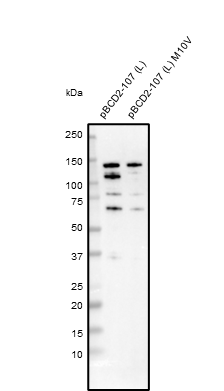
**

**Fig. S3** flCDKL5 production profiles with pBCD-107 (L) plasmids. Anti-FLAG Western blot to detect the C-terminal extremities of 107 (L) and 107 (L) M10V translation products in *P. haloplanktis* TAC125 lysates after recombinant expression.

**
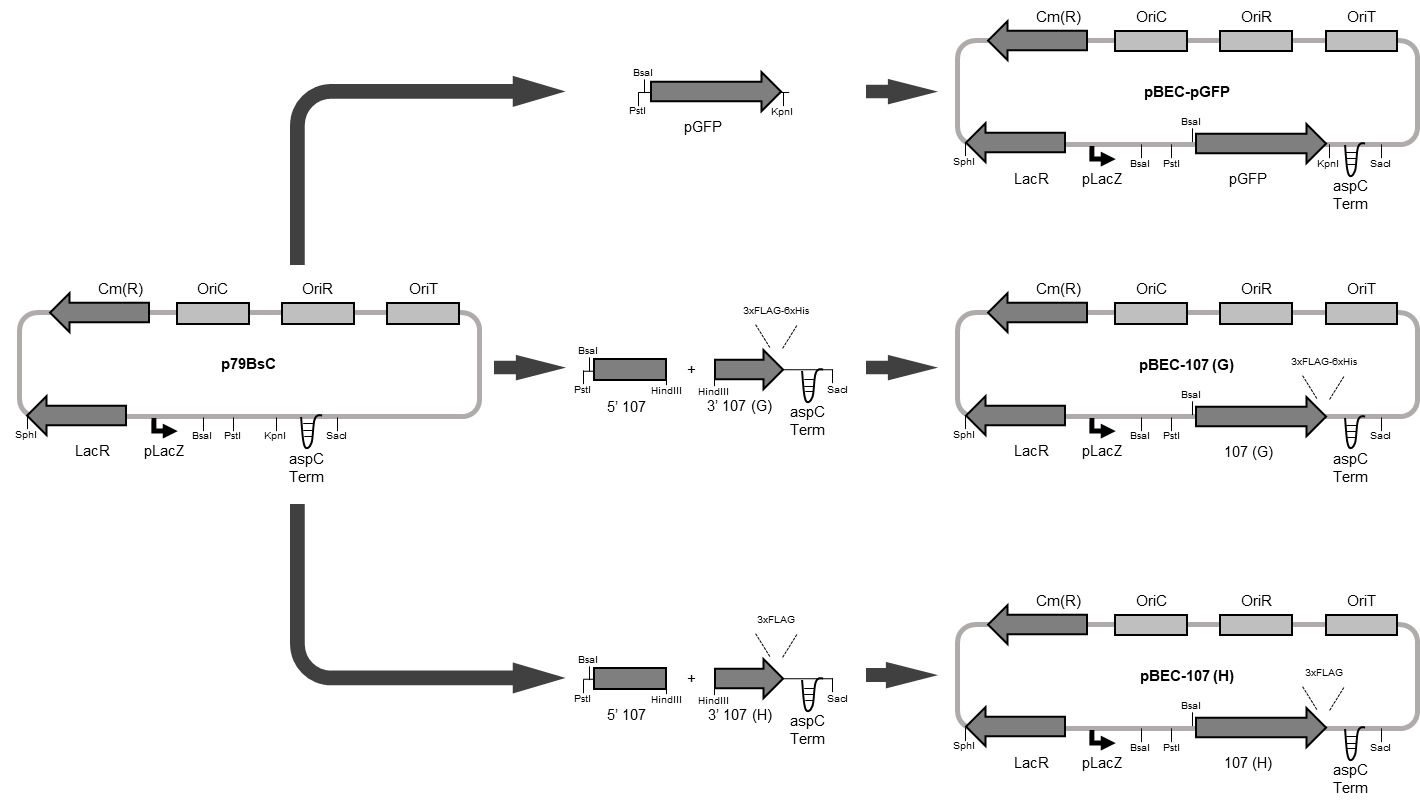
**

**Fig. S4** Development of Bicistronic Entry Clones. The ancestral plasmid p79BsC (left) harbors a BsaI and PstI restriction sites downstream of the pLacZ promoters. pBEC plasmids (right) are generated by cloning the GOIs with PstI and BsaI sites at their 5’ extremities (middle). The resulting pBEC vectors possess two oppositely oriented BsaI sites between the pLacZ promoter and the GOIs so as to allow the scarless cloning of any desired DNA sequence. pBEC-pGFP allowed the study of BCD configurations using pGFP as a reporter, while pBEC-107 (G) and pBEC-107 (H) were used to develop flCDKL5-based BCDs. The first includes a gene encoding flCDKL5 with C-terminal 3xFLAG-6xHis tags, while the latter harbors only a C-terminal 3xFLAG tag.

**
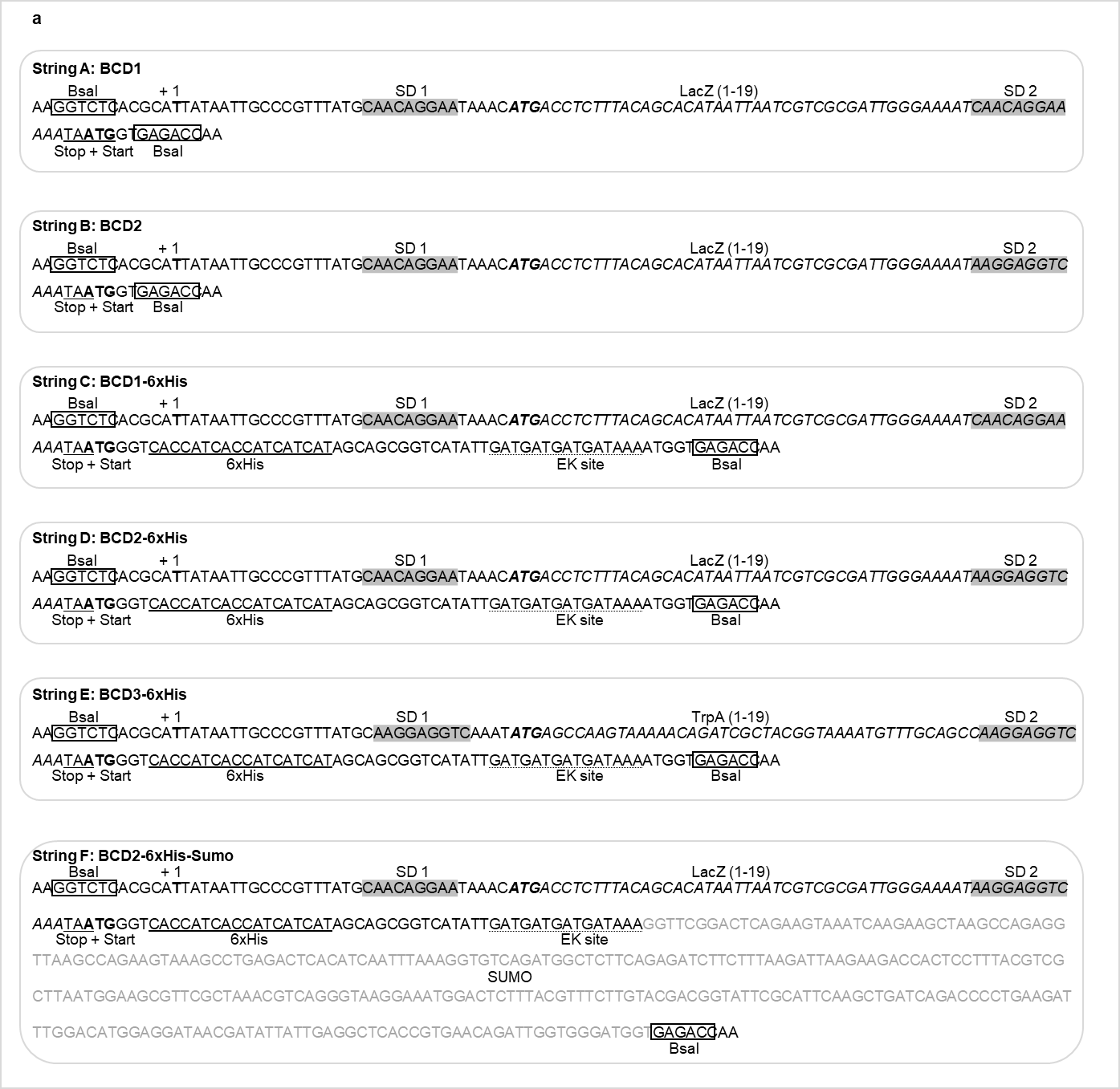
**

**
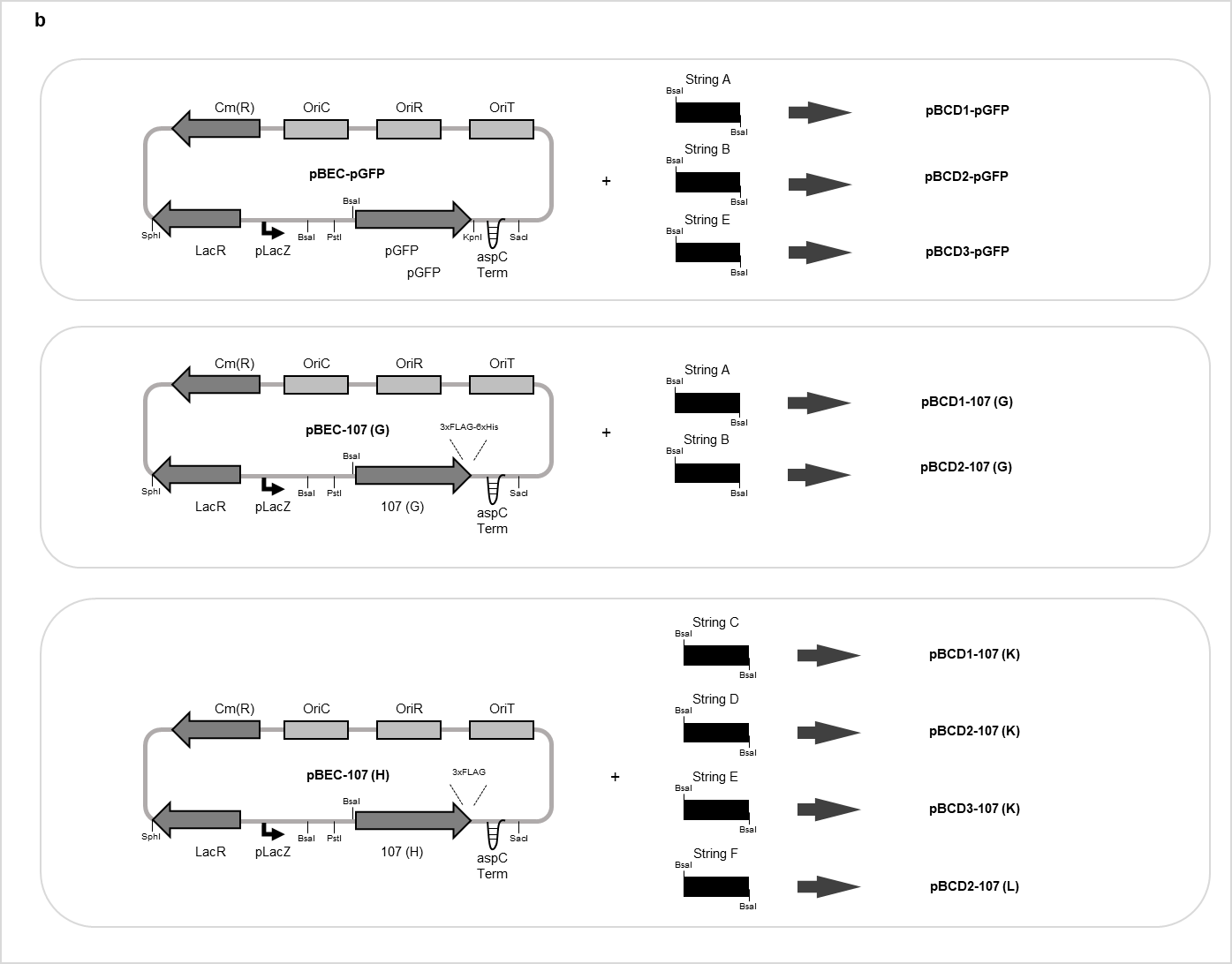
**

**Fig. S5** Development of Bicistronic Designs. **a** Sequences of DNA strings cloned between the pLacZ promoter and the GOIs. Each String harbors two divergent BsaI sites at the extremities (with boxes), the 5’ UTR of p79C (+1 indicates the mRNA start), various combinations of SD1 and SD2 sequences (grey boxes), Leader peptides encoding genes (either LacZ or TrpA-derived sequences in italics) and partially overlapped stop and start codons (TA**ATG**) to trigger the translational coupling. Strings C-F also present sequences encoding different tags (6xHis and Sumo) and a protease recognition site (enterokinase, indicated as EK site) that are in frame with the GOIs after cloning. **b** Cloning schemes to generate BCDs. Different combinations of BECs and strings led to the genesis of all the bicistronic configurations tested in this work (BCD1-3) and differently tagged flCDKL5 variants (107 (G, K, L)).

**
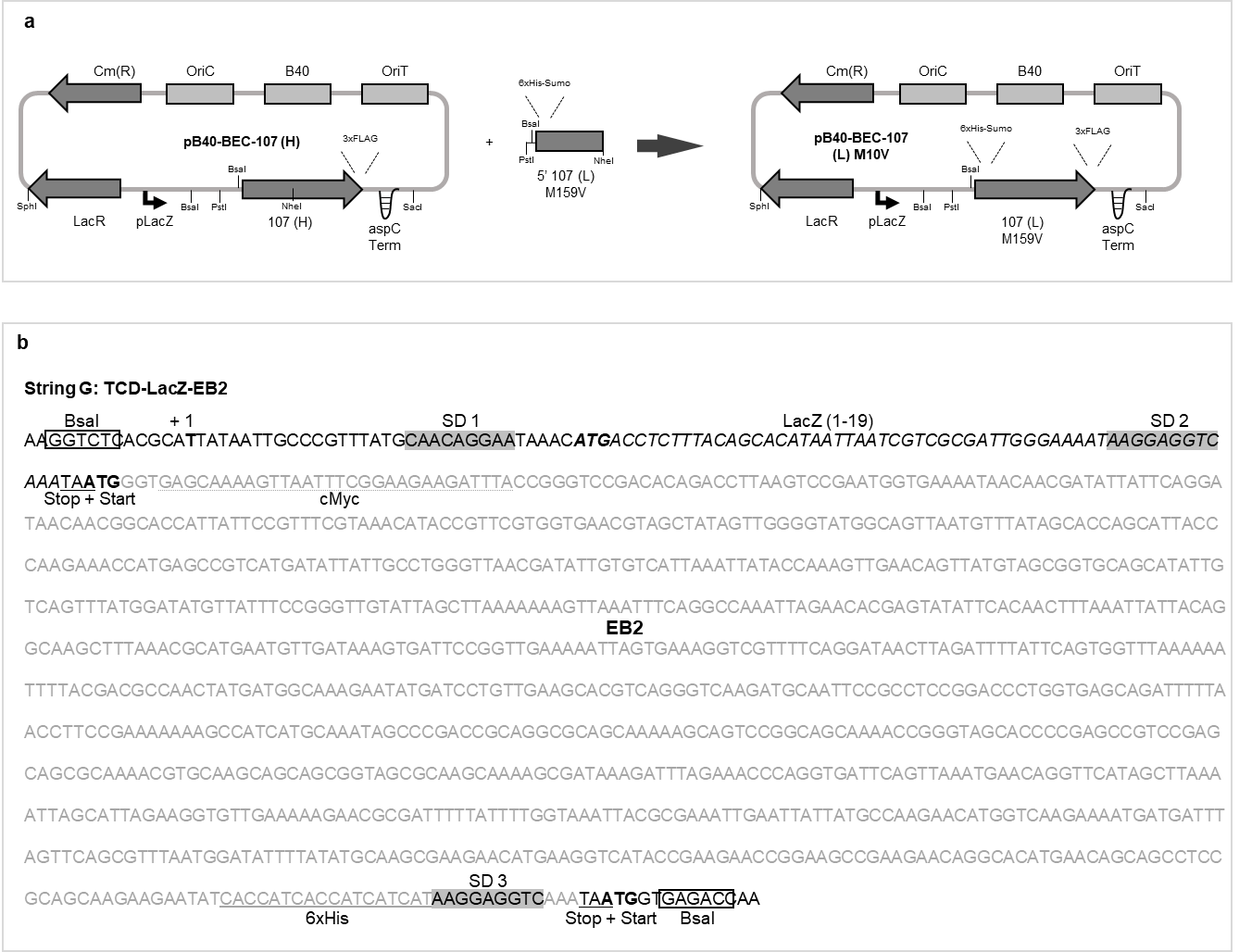
**

**Fig. S6** Development of Tricistronic Designs. **a** Construction of pB40-BEC-107 (L) M10V. pB40-BEC-107 (H) was converted into pB40-BEC-107 (L) M10V by replacing the 5’ half of the flCDKL5 gene. **b** String G sequence to be cloned into pB40-BEC-107 (L) M10V to generate Tricistronic designs (TCDs). String G includes two BsaI sites at its extremities (with boxes), the p79C 5’ UTR (+1 indicates the transcription start), three Shine Dalgarno sequences (grey boxes), the sequence encoding the LacZ (1-19) leader peptide shown in italics and the ORF of EB2 with an N-terminal cMyc and a C-terminal 6xHis tags indicated in light grey. The translational coupling between the three cistrons (Leader, EB2 and flCDKL5) is achieved through the overlap of a stop and start codons (TA**ATG**).

**Nucleotide sequences of flCDKL5 constructs cloned into pMAV and p79C**

>107(B)

catatgggtgatgcggcacaacctgcacgtcgtgctcgtcgcactaaattagctgcatacgcacgtaaagcagcacgtcaagctcgtgctggtggtggtggcagcaaaatccctaacattggtaatgtaatgaacaaattcgaaattcttggtgtagtaggtgaaggtgcttacggtgttgtacttaaatgtcgtcacaaagagacacacgaaatagtagcgatcaagaagttcaaagattctgaagagaatgaagaagtaaaagaaacaacactacgtgaactaaaaatgctgcgtacactaaagcaagaaaacattgttgaattaaaagaggcttttcgacgtcgtggtaaactatatctggttttcgaatacgtagagaaaaacatgttggaattactagaagagatgccaaacggtgttccaccagaaaaagtaaaatcatatatctaccaacttatcaaagctatccattggtgtcataaaaacgacatagttcacagggacattaaacctgagaacctacttatcagccataacgacgtacttaaactttgtgatttcggctttgctcgtaatctaagcgaaggcaacaatgcaaactatacagaatacgtagctactcgttggtatcgcagccctgaacttctactaggtgcaccttatggaaaatctgtagatatgtggtcagtaggatgtatccttggtgaactttctgacggccagcctcttttcccaggtgagtcagaaatcgaccaacttttcacaattcaaaaggtacttggtccattaccttctgaacaaatgaagcttttttactcaaatccacgttttcacggccttcgtttccccgctgtcaaccacccacaatcacttgaacgtcgttacttaggtatactaaactcagttctcctggatcttatgaaaaatttattaaaacttgatccagctgatcgctacctcactgaacagtgtctaaatcatccgacttttcagacacaacgtttattagaccgctcacctagccgttctgcaaagcgtaagccttaccacgtagaaagctcaactctcagcaaccgtaatcaagcaggcaagtctactgcacttcaatctcaccatcgcagcaacagcaaagacatccagaatctttctgttggtcttccacgtgccgatgaaggcttacctgcaaacgaatcttttctaaacggcaatcttgcaggcgctagtctttctccacttcacacaaaaacttatcaagctagcagccaaccaggaagtacttcaaaagatcttactaataataatatcccacacctactttctccgaaagaggcaaaatcaaagactgagtttgatttcaacatcgaccctaaaccttcagaaggtccaggtacaaagtaccttaaaagtaatagccgttcacagcaaaaccgtcattctttcatggaatcaagccaatctaaagctggtacattacaaccaaacgaaaaacaatctcgccattcatacattgataccatcccacagagttctcgctctccatcttaccgcactaaagctaaatctcatggtgcactaagcgactctaagagtgtatcaaacctttcagaagctcgtgctcagatcgcagagccgtctacgagtcgttattttccttcttcttgcttagacttaaactcacctacttctccaacgccaactcgtcactcagatacacgtacgcttctgtctccaagtggtcgtaacaaccgcaatgagggtactctggatagccgtcgtacgacaactcgtcacagtaaaactatggaagaactaaaacttccagaacacatggactctagccattctcactctcttagtgctcctcacgagtctttctcatacggtttaggttacacttcaccattttctagccagcaacgtcctcaccgtcattctatgtacgttacacgtgacaaagtccgtgctaaaggtttggacggttcactttctattgggcaaggtatggctgctcgtgcaaactctctacaactactatcaccacaacctggcgaacaattacctccagaaatgacagttgctcgttcttctgtaaaagaaacgtcacgtgaaggtacatcatcattccatacacgtcagaagagcgagggtggtgtttaccacgacccacactctgacgatggtactgcaccaaaagaaaatagacatctatacaacgatccagtaccacgtcgtgttggtagcttctaccgcgttccttcacctcgtcctgataacagctttcacgaaaataacgtttcaactcgtgttagttctctacctagtgaatcatcttctggcactaaccatagcaaacgtcaaccagcttttgacccttggaagtcacctgaaaacatcagccattcagaacagctaaaggaaaaagagaaacaaggttttttccgtagtatgaagaaaaagaaaaaaaaaagccaaactgttccaaactctgattcaccagatcttcttactcttcagaaaagcattcactctgcttctactccaagctcgcgtccgaaggaatggcgtcctgagaagatcagcgacttacaaactcaatctcaacctctaaaatctttacgtaaacttcttcatttatcttctgcatctaatcaccctgctagttctgatcctcgtttccaaccattaacagcacaacagactaaaaactcgttttctgaaatccgcattcatccactgtcacaggcttcaggtggtagttctaacatccgtcaggaaccagcacctaaaggtcgtcctgcacttcagctacctggtcaaatggacccaggttggcatgttagtagtgttactcgtagcgctactgagggtccatcatactctgaacaacttggcgcaaaatcaggtccaaatggccatccatacaaccgtactaatcgcagtcgtatgccaaacttaaatgatttaaaggaaactgctcttggcggcggtggctctgagaatttatatttccaaggtgattacaaggatcacgacggcgactataaagaccatgacattgattataaagatgatgatgataaagacggtgctcctcaccatcaccatcatcattgataatctagagaattc

>107(G)

catatgggtgatgcggcacaacctgcacgtcgtgctcgtcgcactaaattagctgcatacgcacgtaaagcagcacgtcaagctcgtgctggtggtggtggcagcaaaatccctaacattggtaatgtaatgaacaaattcgaaattcttggtgtagtaggtgaaggtgcttacggtgttgtacttaaatgtcgtcacaaagagacacacgaaatagtagcgatcaagaagttcaaagattctgaagagaatgaagaagtaaaagaaacaacactacgtgaactaaaaatgctgcgtacactaaagcaagaaaacattgttgaattaaaagaggcttttcgacgtcgtggtaaactatatctggttttcgaatacgtagagaaaaacatgttggaattactagaagagatgccaaacggtgttccaccagaaaaagtaaaatcatatatctaccaacttatcaaagctatccattggtgtcataaaaacgacatagttcacagggacattaaacctgagaacctacttatcagccataacgacgtacttaaactttgtgatttcggctttgctcgtaatctaagcgaaggcaacaatgcaaactatacagaatacgtagctactcgttggtatcgcagccctgaacttctactaggtgcaccttatggaaaatctgtagatatgtggtcagtaggatgtatccttggtgaactttctgacggccagcctcttttcccaggtgagtcagaaatcgaccaacttttcacaattcaaaaggtacttggtccattaccttctgaacaaatgaagcttttttactcaaatccacgttttcacggccttcgtttccccgctgtcaaccacccacaatcacttgaacgtcgttacttaggtatactaaactcagttctcctggatcttatgaaaaatttattaaaacttgatccagctgatcgctacctcactgaacagtgtctaaatcatccgacttttcagacacaacgtttattagaccgctcacctagccgttctgcaaagcgtaagccttaccacgtagaaagctcaactctcagcaaccgtaatcaagcaggcaagtctactgcacttcaatctcaccatcgcagcaacagcaaagacatccagaatctttctgttggtcttccacgtgccgatgaaggcttacctgcaaacgaatcttttctaaacggcaatcttgcaggcgctagtctttctccacttcacacaaaaacttatcaagctagcagccaaccaggaagtacttcaaaagatcttactaataataatatcccacacctactttctccgaaagaggcaaaatcaaagactgagtttgatttcaacatcgaccctaaaccttcagaaggtccaggtacaaagtaccttaaaagtaatagccgttcacagcaaaaccgtcattctttcatggaatcaagccaatctaaagctggtacattacaaccaaacgaaaaacaatctcgccattcatacattgataccatcccacagagttctcgctctccatcttaccgcactaaagctaaatctcatggtgcactaagcgactctaagagtgtatcaaacctttcagaagctcgtgctcagatcgcagagccgtctacgagtcgttattttccttcttcttgcttagacttaaactcacctacttctccaacgccaactcgtcactcagatacacgtacgcttctgtctccaagtggtcgtaacaaccgcaatgagggtactctggatagccgtcgtacgacaactcgtcacagtaaaactatggaagaactaaaacttccagaacacatggactctagccattctcactctcttagtgctcctcacgagtctttctcatacggtttaggttacacttcaccattttctagccagcaacgtcctcaccgtcattctatgtacgttacacgtgacaaagtccgtgctaaaggtttggacggttcactttctattgggcaaggtatggctgctcgtgcaaactctctacaactactatcaccacaacctggcgaacaattacctccagaaatgacagttgctcgttcttctgtaaaagaaacgtcacgtgaaggtacatcatcattccatacacgtcagaagagcgagggtggtgtttaccacgacccacactctgacgatggtactgcaccaaaagaaaatagacatctatacaacgatccagtaccacgtcgtgttggtagcttctaccgcgttccttcacctcgtcctgataacagctttcacgaaaataacgtttcaactcgtgttagttctctacctagtgaatcatcttctggcactaaccatagcaaacgtcaaccagcttttgacccttggaagtcacctgaaaacatcagccattcagaacagctaaaggaaaaggagaagcaaggttttttccgtagtatgaagaagaagaagaagaagagccaaactgttccaaactctgattcaccagatcttcttactcttcagaaaagcattcactctgcttctactccaagctcgcgtccgaaggaatggcgtcctgagaagatcagcgacttacaaactcaatctcaacctctaaaatctttacgtaaacttcttcatttatcttctgcatctaatcaccctgctagttctgatcctcgtttccaaccattaacagcacaacagactaaaaactcgttttctgaaatccgcattcatccactgtcacaggcttcaggtggtagttctaacatccgtcaggaaccagcacctaaaggtcgtcctgcacttcagctacctggtcaaatggacccaggttggcatgttagtagtgttactcgtagcgctactgagggtccatcatactctgaacaacttggcgcaaaatcaggtccaaatggccatccatacaaccgtactaatcgcagtcgtatgccaaacttaaatgatttaaaggaaactgctcttggcggcggtggctctgagaatttatatttccaaggtgattacaaggatcacgacggcgactataaagaccatgacattgattataaagatgatgatgataaagacggtgctcctcaccatcaccatcatcattgataaccatgggaattc

>107(H)

catatgggtgatgcggcacaacctgcacgtcgtgctcgtcgcactaaattagctgcatacgcacgtaaagcagcacgtcaagctcgtgctggtggtggtggcagcaaaatccctaacattggtaatgtaatgaacaaattcgaaattcttggtgtagtaggtgaaggtgcttacggtgttgtacttaaatgtcgtcacaaagagacacacgaaatagtagcgatcaagaagttcaaagattctgaagagaatgaagaagtaaaagaaacaacactacgtgaactaaaaatgctgcgtacactaaagcaagaaaacattgttgaattaaaagaggcttttcgacgtcgtggtaaactatatctggttttcgaatacgtagagaaaaacatgttggaattactagaagagatgccaaacggtgttccaccagaaaaagtaaaatcatatatctaccaacttatcaaagctatccattggtgtcataaaaacgacatagttcacagggacattaaacctgagaacctacttatcagccataacgacgtacttaaactttgtgatttcggctttgctcgtaatctaagcgaaggcaacaatgcaaactatacagaatacgtagctactcgttggtatcgcagccctgaacttctactaggtgcaccttatggaaaatctgtagatatgtggtcagtaggatgtatccttggtgaactttctgacggccagcctcttttcccaggtgagtcagaaatcgaccaacttttcacaattcaaaaggtacttggtccattaccttctgaacaaatgaagcttttttactcaaatccacgttttcacggccttcgtttccccgctgtcaaccacccacaatcacttgaacgtcgttacttaggtatactaaactcagttctcctggatcttatgaaaaatttattaaaacttgatccagctgatcgctacctcactgaacagtgtctaaatcatccgacttttcagacacaacgtttattagaccgctcacctagccgttctgcaaagcgtaagccttaccacgtagaaagctcaactctcagcaaccgtaatcaagcaggcaagtctactgcacttcaatctcaccatcgcagcaacagcaaagacatccagaatctttctgttggtcttccacgtgccgatgaaggcttacctgcaaacgaatcttttctaaacggcaatcttgcaggcgctagtctttctccacttcacacaaaaacttatcaagctagcagccaaccaggaagtacttcaaaagatcttactaataataatatcccacacctactttctccgaaagaggcaaaatcaaagactgagtttgatttcaacatcgaccctaaaccttcagaaggtccaggtacaaagtaccttaaaagtaatagccgttcacagcaaaaccgtcattctttcatggaatcaagccaatctaaagctggtacattacaaccaaacgaaaaacaatctcgccattcatacattgataccatcccacagagttctcgctctccatcttaccgcactaaagctaaatctcatggtgcactaagcgactctaagagtgtatcaaacctttcagaagctcgtgctcagatcgcagagccgtctacgagtcgttattttccttcttcttgcttagacttaaactcacctacttctccaacgccaactcgtcactcagatacacgtacgcttctgtctccaagtggtcgtaacaaccgcaatgagggtactctggatagccgtcgtacgacaactcgtcacagtaaaactatggaagaactaaaacttccagaacacatggactctagccattctcactctcttagtgctcctcacgagtctttctcatacggtttaggttacacttcaccattttctagccagcaacgtcctcaccgtcattctatgtacgttacacgtgacaaagtccgtgctaaaggtttggacggttcactttctattgggcaaggtatggctgctcgtgcaaactctctacaactactatcaccacaacctggcgaacaattacctccagaaatgacagttgctcgttcttctgtaaaagaaacgtcacgtgaaggtacatcatcattccatacacgtcagaagagcgagggtggtgtttaccacgacccacactctgacgatggtactgcaccaaaagaaaatagacatctatacaacgatccagtaccacgtcgtgttggtagcttctaccgcgttccttcacctcgtcctgataacagctttcacgaaaataacgtttcaactcgtgttagttctctacctagtgaatcatcttctggcactaaccatagcaaacgtcaaccagcttttgacccttggaagtcacctgaaaacatcagccattcagaacagctaaaggaaaaggagaagcaaggttttttccgtagtatgaagaagaagaagaagaagagccaaactgttccaaactctgattcaccagatcttcttactcttcagaaaagcattcactctgcttctactccaagctcgcgtccgaaggaatggcgtcctgagaagatcagcgacttacaaactcaatctcaacctctaaaatctttacgtaaacttcttcatttatcttctgcatctaatcaccctgctagttctgatcctcgtttccaaccattaacagcacaacagactaaaaactcgttttctgaaatccgcattcatccactgtcacaggcttcaggtggtagttctaacatccgtcaggaaccagcacctaaaggtcgtcctgcacttcagctacctggtcaaatggacccaggttggcatgttagtagtgttactcgtagcgctactgagggtccatcatactctgaacaacttggcgcaaaatcaggtccaaatggccatccatacaaccgtactaatcgcagtcgtatgccaaacttaaatgatttaaaggaaactgctcttggcggcggtggctctgagaatttatatttccaaggtgattacaaggatcacgacggcgactataaagaccatgacattgattataaagatgatgatgataaagactgataaccatgggaattc

>EB2

catatgggtcatcatcaccatcatcatggatcaactagtggttctggtccgggtccgacacagaccttaagtccgaatggtgaaaataacaacgatattattcaggataacaacggcaccattattccgtttcgtaaacataccgttcgtggtgaacgtagctatagttggggtatggcagttaatgtttatagcaccagcattacccaagaaaccatgagccgtcatgatattattgcctgggttaacgatattgtgtcattaaattataccaaagttgaacagttatgtagcggtgcagcatattgtcagtttatggatatgttatttccgggttgtattagcttaaaaaaagttaaatttcaggccaaattagaacacgagtatattcacaactttaaattattacaggcaagctttaaacgcatgaatgttgataaagtgattccggttgaaaaattagtgaaaggtcgttttcaggataacttagattttattcagtggtttaaaaaattttacgacgccaactatgatggcaaagaatatgatcctgttgaagcacgtcagggtcaagatgcaattccgcctccggaccctggtgagcagatttttaaccttccgaaaaaaagccatcatgcaaatagcccgaccgcaggcgcagcaaaaagcagtccggcagcaaaaccgggtagcaccccgagccgtccgagcagcgcaaaacgtgcaagcagcagcggtagcgcaagcaaaagcgataaagatttagaaacccaggtgattcagttaaatgaacaggttcatagcttaaaattagcattagaaggtgttgaaaaagaacgcgatttttattttggtaaattacgcgaaattgaattattatgccaagaacatggtcaagaaaatgatgatttagttcagcgtttaatggatattttatatgcaagcgaagaacatgaaggtcataccgaagaaccggaagccgaagaacaggcacatgaacagcagcctccgcagcaagaagaatattaaggatcc
